# Global, regional, and national burden of ovarian cancer and uterine cancer attributable to high BMI, 1990-2021: analysis of data from the global burden of disease study 2021

**DOI:** 10.3389/fonc.2026.1821189

**Published:** 2026-05-28

**Authors:** Xi Wang, Lin Ma

**Affiliations:** The Reproductive Medical Center, The Seventh Affiliated Hospital of Sun Yat-sen University, Shenzhen, China

**Keywords:** attributable to high BMI, early-onset and late-onset cancer, global burden of disease, ovarian cancer, uterine cancer

## Abstract

**Background:**

High body mass index (BMI) has been identified as a critical factor contributing to the occurrence of ovarian cancer and uterine cancer. This study intended to analyze the global epidemiological trends of ovarian cancer and uterine cancer attributable to high BMI.

**Methods:**

Data on early-onset (<50 years old) and late-onset (≥50 years old) ovarian cancer and uterine cancer attributable to high BMI from 1990 to 2021 were extracted from the Global Burden of Disease (GBD) 2021. Mortality, disability-adjusted life-years (DALYs), and average annual percentage change (AAPC) were applied to assess the disease burden. Future trends in mortality and DALYs were projected using the Bayesian age-period-cohort analysis.

**Results:**

From 1990 to 2021, the DALYs of early-onset and late-onset ovarian cancer and uterine cancer attributable to high BMI all showed an upward trend, with AAPCs of 1.363 (95%CI: 1.336 to 1.400), 0.405 (95%CI: 0.382 to 0.431), 0.560 (95%CI: 0.490 to 0.637), and 0.300 (95%CI: 0.254 to 0.342), respectively. The mortality outcomes for ovarian cancer and uterine cancer were similar to those observed in DALYs. The age-standardized rates of mortality and DALYs for late-onset ovarian cancer and uterine cancer were significantly higher than those for their early-onset cases. Among regions of different socio-demographic index (SDI), the mortality and DALYs of early-onset and late-onset ovarian cancer exhibited a declining trend in high-SDI regions, while they showed an increasing trend in other SDI regions. For uterine cancer, the mortality and DALYs presented a downward trend in high-middle-SDI regions. Furthermore, the mortality and DALYs for ovarian cancer and uterine cancer are projected to continue rising from 2022 to 2050.

**Conclusions:**

The global burden of early-onset and late-onset ovarian cancer and uterine cancer attributable to high BMI has shown an upward trend from 1990 to 2021 and is projected to continue rising in the future.

## Introduction

Ovarian cancer and uterine cancer are common malignant tumors among women worldwide (1). Ovarian cancer is the second leading cause of death from gynecological cancers, following cervical cancer (2). Globally, there were approximately 324,000 new cases (1.6% of all cancers) and 206,000 deaths of ovarian cancer (2.1%) in 2022, as well as 420,000 new cases (2.1%) and 97,000 deaths of uterine cancer (1.0%) (2). Despite advances in screening, surgery, and treatment methods, the survival rate for ovarian cancer has remained relatively stable over the past few decades, with a 5-year survival rate of only 47% after diagnosis (3). The epidemiological characteristics of different cancers may exhibit significant geographical variations and temporal changes (4, 5). Analyzing the epidemiological features of specific ovarian cancer and uterine cancer is crucial for understanding their public health implications.

High body mass index (BMI) has been reported as a significant factor contributing to deaths and disability-adjusted life years (DALYs) from ovarian cancer and uterine cancer (6, 7). The link between a high BMI (e.g., overweight, obesity) and the occurrence of uterine cancer is associated with chronic inflammation, high estrogenism, and insulin resistance (8, 9). The association between BMI and ovarian cancer is more complex, potentially involving hormonal imbalance, chronic inflammation, insulin resistance, genetic factors, and menopausal status (10, 11). The disease burden attributable to high BMI also varies significantly across different age groups, with the disease burden related to high BMI increasing relatively rapidly among elderly women (12, 13). Population aging is the primary driver of the increased disease burden correlated to high BMI (14), but the disease burden attributable to high BMI among younger individuals has also risen significantly (15). However, the trends in the disease burden of ovarian cancer and uterine cancer attributable to high BMI among different age groups remain unclear. The age of 50 is commonly used to distinguish between early-onset (<50 years old) and late-onset (≥50 years old) cancers (16). Previous studies have reported significant differences in genetic susceptibility, pathological characteristics, and prognostic factors between early-onset and late-onset ovarian cancer (17–19), as well as uterine cancer (20, 21). Additionally, the disease burden of early-onset and late-onset ovarian cancer and uterine cancer exhibits distinct trends and significant regional variations (5, 22, 23). The Global Burden of Disease (GBD) Study 2021 represents a large-scale epidemiological database that offers comprehensive epidemiological data encompassing 204 countries and regions, 371 disease entities, and 88 attributable risk factors from 1990 to 2021. Therefore, based on data from the GBD 2021, this study intended to explore the global trends in mortality and DALYs for early-onset and late-onset ovarian cancer and uterine cancer attributable to high BMI.

## Methods

### Data sources and patients

Data employed in this study were obtained from the GBD 2021. The GBD study is a publicly accessible epidemiological database, which provides the latest epidemiological data estimates for 371 diseases and 88 risk factors in 21 GBD regions and 204 countries/regions from 1990 to 2021 (https://ghdx.healthdata.org/gbd-2021). The GBD visualization platform (https://vizhub.healthdata.org/gbd-results/) was applied to extract mortality and DALYs data and their 95% confidence intervals (CI) for ovarian cancer and uterine cancer attributable to high BMI from 1990 to 2021. High BMI in GBD was defined as BMI ≥ 25kg/m^2^ (24). Ovarian cancer and uterine cancer were identified by the International Classification of Diseases-10th (ICD-10) codes: ovarian cancer (ICD-10: C56-C56.9, D27-D27.9, D39.1); uterine cancer (ICD-10: C54-C54.9, D07.0-D07.2, D26.1-D26.9) (25). In this analysis, ovarian cancer and uterine cancer represent the broader GBD category called ovarian cancer and uterine cancer.

Since disease burden data for risk factors are only available for individuals aged 20 years and above, this analysis included patients aged ≥20 years. Patients were grouped according to each 5-year age group: 20–24 years, 25–29 years, 30–34 years, 35–39 years, 40–44 years, 45–49 years, 50–54 years, 55–59 years, 60–64 years, 65–69 years, 70–74 years, 75–79 years, 80–84 years, 85–89 years, 90–94 years, and ≥95 years. The age of 50 is commonly used to distinguish between early-onset and late-onset tumors (16, 26), hence, the classification in this study was: early-onset cancers (20–49 years old), late-onset cancers (≥50 years old). The socio-demographic index (SDI) is an indicator for assessing the development level of various regions, with a higher SDI value representing a higher level of development. Countries and regions are categorized into five different groups based on their SDI values: low-SDI, medium-low-SDI, medium-SDI, medium-high-SDI, and high-SDI (27). This study was exempt from ethics review and informed consent procedures as it utilized publicly available anonymized data obtained from the GBD database.

### Mortality, DALYs, and future trends

The age-standardized rate (ASR) per 100,000 people was utilized to represent mortality and DALYs: age-standardized mortality rates (ASMR) and age-standardized rates of DALYs (ASDR). ASR was calculated according to the following formula (28): ASR = $\sum_{i=1}^{N}\alpha_{i}\omega_{i}/\sum_{i=1}^{N}\omega_{i}$, where i is the age group (in 5-year intervals), N is the total number of age groups, α_i_ is the age-specific rate, and ω_i_ is the weight derived from the standard population corresponding to each of the respective age groups.

The trends in mortality (ASMR) and DALYs (ASDR) from 1990 to 2021 were analyzed using the average annual percentage change (AAPC) with 95% confidence interval (CI) through the Joinpoint regression model. If the AAPC and its 95% CI are above or below zero, it indicates a trend of ASR increasing or decreasing over time. The joinpoint regression model is a commonly used method for analyzing temporal trends in disease (29). Based on the temporal distribution characteristics of the disease, it divides the study period into multiple segments and performs trend fitting and optimization for each segment. Using a grid search method, it establishes all possible joinpoints for segmented functions within the intervals, calculates the corresponding mean squared error (MSE), and selects the grid point with the smallest MSE as the joinpoint.

Future trends in mortality and DALYs from 2022 to 2050 were projected using the Bayesian age-period-cohort analysis with integrated nested Laplace approximation. This Bayesian model integrates age effects (risk changes with age), period effects (impact at specific time points), and cohort effects (risk attributes related to birth cohorts), and predicts future trends through a comprehensive analysis of past data.

### Statistical analysis

The ASMR and ASDR of ovarian cancer and uterine cancer (early-onset and late-onset) attributable to high BMI in 1990 and 2021 were calculated, as well as the AAPC from 1990 to 2021. The future trends of ASMR and ASDR for ovarian cancer and uterine cancer from 2022 to 2050 were analyzed. The analysis and future prediction of ASMR and ASDR were conducted using R version 4.5.1 software (Institute for Statistics and Mathematics, Vienna, Austria), whereas the trend analysis and AAPC calculation were carried out using Jointpoint 5.4.0.0 software (National Cancer Institute, Bethesda, MD, USA). Statistical significance was set at P < 0.05.

## Results

### Global mortality and DALYs of ovarian cancer and uterine cancer attributable to high BMI

**Table 1** shows the mortality and DALYs of ovarian cancer and uterine cancer attributable to high BMI globally and in different SDI regions. Globally, 2,022 (95%CI: 461 to 3,628) cases of early-onset ovarian cancer died in 2021, and the ASMR showed an increasing trend from 1990 to 2021 [AAPC: 1.288 (95%CI: 1.256 to 1.325)]. For DALYs, 99,915 (95%CI: 22,668 to 179,627) cases of early-onset ovarian cancer occurred DALYs in 2021. The ASDR of early-onset ovarian cancer also presented an increasing trend from 1990 to 2021 [AAPC: 1.363 (95%CI: 1.336 to 1.400)]. Among different SDI regions, DALYs of early-onset ovarian cancer showed a declining trend in high-SDI regions [AAPC: -0.174 (95%CI: -0.216 to -0.133)] from 1990 to 2021, while the largest increase in DALYs occurred in low-middle SDI regions [AAPC: 4.465 (95%CI: 4.429 to 4.499)]. The mortality of early-onset ovarian cancer in different SDI regions was similar to that of DALYs.

**Table 1:** Global mortality and DALYs of ovarian cancer and uterine cancer attributable to high BMI from 1990 to 2021.

Cancers	Variables	Number in 1990 (95% CI)	ASR in 1990 (95% CI)	Number in 2021 (95% CI)	ASR in 2021 (95% CI)	AAPC of ASR (95% CI)	*P*
Early-onset ovarian cancer	DALYs						
	Global	35645 (5647 to 68468)	3.91 (0.64 to 7.48)	99915 (22668 to 179627)	5.97 (1.35 to 10.73)	1.363 (1.336 to 1.400)	<0.001
	High SDI	14630 (2813 to 27422)	7.58 (1.46 to 14.2)	17361 (4220 to 30597)	7.21 (1.75 to 12.71)	-0.174 (-0.216 to -0.133)	<0.001
	High-middle SDI	11970 (2077 to 23158)	6.06 (1.09 to 11.69)	23400 (5321 to 42571)	7.38 (1.67 to 13.43)	0.640 (0.589 to 0.702)	<0.001
	Middle SDI	6165 (512 to 12606)	2.16 (0.21 to 4.4)	35003 (7997 to 64475)	6.27 (1.43 to 11.55)	3.499 (3.467 to 3.532)	<0.001
	Low-middle SDI	2186 (107 to 4649)	1.25 (0.08 to 2.64)	18706 (3830 to 34514)	4.88 (1.01 to 8.98)	4.465 (4.429 to 4.499)	<0.001
	Low SDI	626 (-21 to 1486)	0.93 (-0.01 to 2.19)	5325 (773 to 10568)	3.05 (0.46 to 6.02)	3.889 (3.856 to 3.920)	<0.001
	Mortality						
	Global	730 (120 to 1398)	0.08 (0.01 to 0.15)	2022 (461 to 3628)	0.12 (0.03 to 0.22)	1.288 (1.256 to 1.325)	<0.001
	High SDI	299 (58 to 562)	0.16 (0.03 to 0.29)	351 (86 to 618)	0.14 (0.04 to 0.25)	-0.252 (-0.292 to -0.210)	<0.001
	High-middle SDI	247 (45 to 477)	0.13 (0.02 to 0.24)	483 (110 to 880)	0.15 (0.03 to 0.27)	0.554 (0.501 to 0.613)	<0.001
	Middle SDI	124 (11 to 252)	0.04 (0 to 0.09)	705 (162 to 1297)	0.13 (0.03 to 0.23)	3.424 (3.387 to 3.464)	<0.001
	Low-middle SDI	45 (3 to 94)	0.03 (0 to 0.05)	374 (78 to 690)	0.1 (0.02 to 0.18)	4.378 (4.343 to 4.411)	<0.001
	Low SDI	13 (0 to 30)	0.02 (0 to 0.05)	106 (16 to 210)	0.06 (0.01 to 0.12)	3.799 (3.767 to 3.827)	<0.001
Late-onset ovarian cancer	DALYs						
	Global	153229 (31721 to 287111)	32.69 (6.76 to 61.24)	377334 (91267 to 671852)	37.3 (9.03 to 66.32)	0.405 (0.382 to 0.431)	<0.001
	High SDI	82112 (17717 to 152976)	62.86 (13.62 to 116.88)	127089 (31880 to 227563)	55.6 (14.08 to 99.08)	-0.422 (-0.457 to -0.385)	<0.001
	High-middle SDI	53419 (11740 to 98377)	43.03 (9.45 to 79.23)	114726 (28299 to 204576)	48.56 (11.97 to 86.54)	0.324 (0.277 to 0.370)	<0.001
	Middle SDI	11812 (1565 to 23241)	9.81 (1.29 to 19.31)	86136 (20714 to 156772)	26.52 (6.38 to 48.25)	3.271 (3.256 to 3.288)	<0.001
	Low-middle SDI	4097 (277 to 8657)	5.93 (0.39 to 12.54)	39200 (8370 to 72622)	23.18 (4.94 to 42.96)	4.496 (4.465 to 4.526)	<0.001
	Low SDI	1501 (25 to 3337)	5.74 (0.03 to 12.8)	9617 (1506 to 18734)	16.13 (2.48 to 31.48)	3.404 (3.389 to 3.420)	<0.001
	Mortality						
	Global	6120 (1263 to 11483)	1.28 (0.26 to 2.41)	15322 (3676 to 27398)	1.46 (0.35 to 2.6)	0.387 (0.367 to 0.410)	<0.001
	High SDI	3502 (749 to 6547)	2.45 (0.53 to 4.57)	5836 (1434 to 10532)	2.24 (0.56 to 4.01)	-0.333 (-0.366 to -0.297)	<0.001
	High-middle SDI	1996 (435 to 3688)	1.57 (0.34 to 2.91)	4612 (1129 to 8242)	1.86 (0.46 to 3.31)	0.480 (0.440 to 0.521)	<0.001
	Middle SDI	419 (54 to 823)	0.36 (0.05 to 0.7)	3127 (739 to 5712)	0.96 (0.23 to 1.76)	3.282 (3.263 to 3.306)	<0.001
	Low-middle SDI	142 (9 to 301)	0.21 (0.01 to 0.45)	1397 (292 to 2599)	0.83 (0.17 to 1.55)	4.506 (4.472 to 4.539)	<0.001
	Low SDI	50 (-1 to 111)	0.19 (-0.01 to 0.44)	326 (48 to 638)	0.56 (0.08 to 1.11)	3.497 (3.483 to 3.509)	<0.001
Early-onset uterine cancer	DALYs						<0.001
	Global	53498 (35988 to 73358)	5.82 (3.92 to 7.97)	114177 (79930 to 152290)	6.81 (4.77 to 9.08)	0.560 (0.490 to 0.637)	<0.001
	High SDI	10327 (7245 to 13814)	5.35 (3.75 to 7.16)	20163 (14572 to 26015)	8.26 (5.97 to 10.66)	1.490 (1.395 to 1.555)	<0.001
	High-middle SDI	20558 (13966 to 28149)	10.25 (6.98 to 14.04)	29261 (20413 to 39474)	9.21 (6.42 to 12.44)	-0.273 (-0.368 to -0.163)	<0.001
	Middle SDI	15529 (9680 to 22247)	5.45 (3.42 to 7.78)	38135 (25499 to 52162)	6.79 (4.54 to 9.29)	0.723 (0.691 to 0.750)	<0.001
	Low-middle SDI	5245 (3475 to 7211)	2.99 (1.99 to 4.1)	19397 (13008 to 26674)	5.09 (3.42 to 6.99)	1.712 (1.691 to 1.730)	<0.001
	Low SDI	1726 (1086 to 2495)	2.54 (1.6 to 3.66)	7022 (4413 to 10344)	4.06 (2.55 to 5.97)	1.545 (1.518 to 1.573)	<0.001
	Mortality						
	Global	1053 (708 to 1442)	0.12 (0.08 to 0.16)	2202 (1535 to 2936)	0.13 (0.09 to 0.17)	0.453 (0.399 to 0.520)	<0.001
	High SDI	196 (138 to 264)	0.1 (0.07 to 0.14)	365 (264 to 467)	0.15 (0.11 to 0.19)	1.298 (1.214 to 1.365)	<0.001
	High-middle SDI	404 (275 to 554)	0.2 (0.14 to 0.28)	555 (386 to 753)	0.17 (0.12 to 0.24)	-0.445 (-0.534 to -0.345)	<0.001
	Middle SDI	310 (194 to 443)	0.11 (0.07 to 0.16)	752 (507 to 1036)	0.13 (0.09 to 0.18)	0.611 (0.574 to 0.642)	<0.001
	Low-middle SDI	106 (70 to 145)	0.06 (0.04 to 0.08)	386 (259 to 528)	0.1 (0.07 to 0.14)	1.645 (1.626 to 1.661)	<0.001
	Low SDI	35 (22 to 51)	0.05 (0.03 to 0.08)	140 (88 to 206)	0.08 (0.05 to 0.12)	1.495 (1.473 to 1.520)	<0.001
Late-onset uterine cancer	DALYs						
	Global	319143 (224866 to 427531)	67.41 (47.49 to 90.28)	765969 (544681 to 1006894)	74.43 (53.03 to 97.78)	0.300 (0.254 to 0.342)	<0.001
	High SDI	120949 (85574 to 161987)	86.59 (61.43 to 115.62)	263993 (188268 to 343022)	110.73 (79.77 to 143.09)	0.800 (0.761 to 0.835)	<0.001
	High-middle SDI	130454 (91699 to 173806)	103.05 (72.47 to 137.3)	240727 (169417 to 318311)	98.95 (69.71 to 130.81)	-0.110 (-0.177 to -0.031)	0.01
	Middle SDI	43768 (29427 to 60819)	36.63 (24.63 to 50.89)	164031 (112768 to 225808)	50.55 (34.76 to 69.57)	1.039 (0.997 to 1.076)	<0.001
	Low-middle SDI	16970 (11595 to 23502)	25.21 (17.22 to 34.92)	73887 (49671 to 101321)	43.96 (29.55 to 60.31)	1.817 (1.791 to 1.840)	<0.001
	Low SDI	6335 (4091 to 9164)	25.12 (16.22 to 36.37)	21985 (14078 to 31954)	38.07 (24.38 to 55.35)	1.362 (1.354 to 1.370)	<0.001
	Mortality						
	Global	12841 (9030 to 17245)	2.67 (1.88 to 3.58)	30933 (21783 to 40832)	2.87 (2.03 to 3.79)	0.219 (0.173 to 0.257)	<0.001
	High SDI	5305 (3714 to 7175)	3.45 (2.43 to 4.65)	11473 (7990 to 15108)	4.17 (2.95 to 5.44)	0.615 (0.579 to 0.647)	<0.001
	High-middle SDI	5024 (3521 to 6714)	3.88 (2.72 to 5.19)	9659 (6718 to 12794)	3.75 (2.61 to 4.96)	-0.081 (-0.154 to 0.011)	0.072
	Middle SDI	1620 (1091 to 2250)	1.39 (0.94 to 1.94)	6147 (4194 to 8483)	1.89 (1.29 to 2.61)	0.991 (0.948 to 1.030)	<0.001
	Low-middle SDI	634 (433 to 880)	0.98 (0.67 to 1.37)	2788 (1872 to 3843)	1.68 (1.13 to 2.32)	1.755 (1.713 to 1.785)	<0.001
	Low SDI	229 (148 to 332)	0.95 (0.61 to 1.38)	807 (516 to 1173)	1.45 (0.93 to 2.11)	1.393 (1.383 to 1.404)	<0.001

DALYs, disability-adjusted life-years; BMI, body mass index; ASR, age-standardized rate; AAPC, average annual percentage change; CI, confidence interval.

For late-onset ovarian cancer, 15,322 (95%CI: 3,676 to 27,398) patients died in 2021, and the ASMR showed an increasing trend from 1990 to 2021 [AAPC: 0.387 (95%CI: 0.367 to 0.410)]. In terms of DALYs, 377,334 (95%CI: 91,267 to 671,852) cases developed DALYs in 2021. The ASDR of late-onset ovarian cancer presented an upward trend from 1990 to 2021 [AAPC: 0.405 (95%CI: 0.382 to 0.431)]. In different SDI regions, DALYs of late-onset ovarian cancer also presented a decreasing trend in high-SDI regions [AAPC: -0.422 (95%CI: -0.457 to -0.385)] from 1990 to 2021, while DALYs increased in low-middle SDI regions [AAPC: 4.496 (95%CI: 4.465 to 4.526)], low SDI regions [AAPC: 3.404 (95%CI: 3.389 to 3.420)], middle SDI regions [AAPC: 3.271 (95%CI: 3.256 to 3.288)], and high-middle SDI regions [AAPC: 0.324 (95%CI: 0.277 to 0.370)].

In terms of early-onset uterine cancer, 2,202 (95%CI: 1,535 to 2,936) cases died in 2021. The ASMR of early-onset uterine cancer exhibited an increasing trend from 1990 to 2021 [AAPC: 0.453 (95%CI: 0.399 to 0.520)]. For DALYs, 114,177 (95%CI: 79,930 to 152,290) cases of early-onset uterine cancer developed DALYs in 2021. The ASDR of early-onset uterine cancer showed an upward trend from 1990 to 2021 [AAPC: 0.560 (95%CI: 0.490 to 0.637)]. In different SDI regions, DALYs of early-onset uterine cancer decreased in high-middle SDI regions [AAPC: -0.273 (95%CI: -0.368 to -0.163)] from 1990 to 2021, whereas the largest increase in DALYs was observed in low-middle SDI regions [AAPC: 1.712 (95%CI: 1.691 to 1.73)].

**Figure 1:**
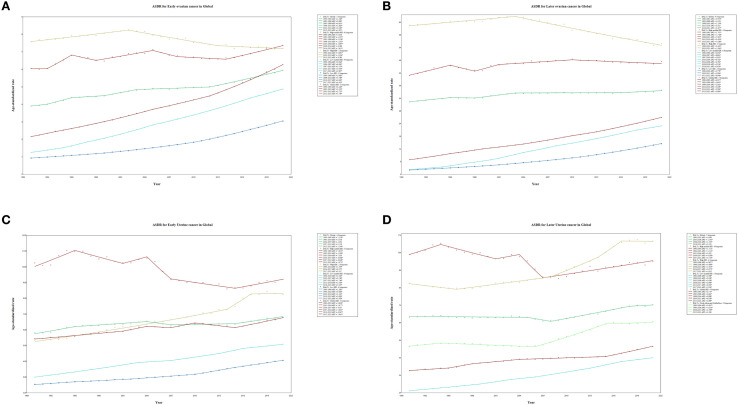
The global DALYs trends of ovarian cancer and uterine cancer attributable to high BMI. **(A)** DALYs trends of early-onset ovarian cancer; **(B)** DALYs trends of late-onset ovarian cancer; **(C)** DALYs trends of early-onset uterine cancer; **(D)** DALYs trends of late-onset uterine cancer. A positive APC value indicates that the ASDR is increasing annually, while a negative APC value indicates that the ASDR is decreasing annually. DALYs, disability-adjusted life-years; ASDR, age-standardized rates of DALYs; BMI, body mass index; APC, annual percentage change.

For late-onset uterine cancer, 30,933 (95%CI: 21,783 to 40,832) patients died in 2021, and the ASMR presented an upward trend from 1990 to 2021 [AAPC: 0.219 (95%CI: 0.173 to 0.257)]. For DALYs, 765,969 (95%CI: 544,681 to 1,006,894) cases of late-onset uterine cancer occurred DALYs in 2021. The ASDR exhibited an increasing trend from 1990 to 2021 [AAPC: 0.300 (95%CI: 0.254 to 0.342)]. Among different SDI regions, the largest increase in DALYs of late-onset uterine cancer from 1990 to 2021 was found in low-middle SDI regions [AAPC: 1.817 (95%CI: 1.791 to 1.840)], while the DALYs decreased in high-middle SDI regions [AAPC: -0.110 (95%CI: -0.177 to -0.031)]. The global DALYs trends of ovarian cancer and uterine cancer attributable to high BMI are displayed in **Figure 1**.

### Mortality and DALYs of ovarian cancer and uterine cancer attributable to high BMI in 21 GBD regions and countries

Among different GBD regions, Central Latin America [ASMR: 0.33 (95%CI: 0.09 to 0.60); ASDR: 16.28 (95%CI: 4.57 to 29.69)] and Eastern Europe [ASMR: 0.31 (95%CI: 0.07 to 0.56); ASDR: 14.86 (95%CI: 3.48 to 27.05)] had the highest ASMR and ASDR for early-onset ovarian cancer in 2021 (**Supplementary Table 1**). The highest rates of ASMR and ASDR for late-onset ovarian cancer in 2021 were in Central Europe [ASMR: 3.79 (95%CI: 0.99 to 6.89); ASDR: 97.25 (95%CI: 25.48 to 177.07)] and Eastern Europe [ASMR: 3.49 (95%CI: 0.90 to 6.13); ASDR: 94.99 (95%CI: 24.72 to 166.35)] (**Supplementary Table 2**). For early-onset uterine cancer, Caribbean [ASMR: 0.57 (95%CI: 0.38 to 0.81); ASDR: 28.92 (95%CI: 19.02 to 40.94)] and Eastern Europe [ASMR: 0.40 (95%CI: 0.26 to 0.53); ASDR: 21.09 (95%CI: 14.26 to 28.57)] had the highest ASMR and ASDR in 2021 (**Supplementary Table 3**). Moreover, the highest rates of ASMR and ASDR for late-onset uterine cancer in 2021 were in Caribbean [ASMR: 7.43 (95%CI: 5.06 to 10.3); ASDR: 190.93 (95%CI: 131.14 to 264.75)], Central Europe [ASMR: 7.02 (95%CI: 4.92 to 9.38); ASDR: 175.25 (95%CI: 124.29 to 233.88)], and High-income North America [ASMR: 6.42 (95%CI: 4.56 to 8.23); ASDR: 176.43 (95%CI: 127.61 to 223.98)] (**Supplementary Table 4**).

**Figure 2:**
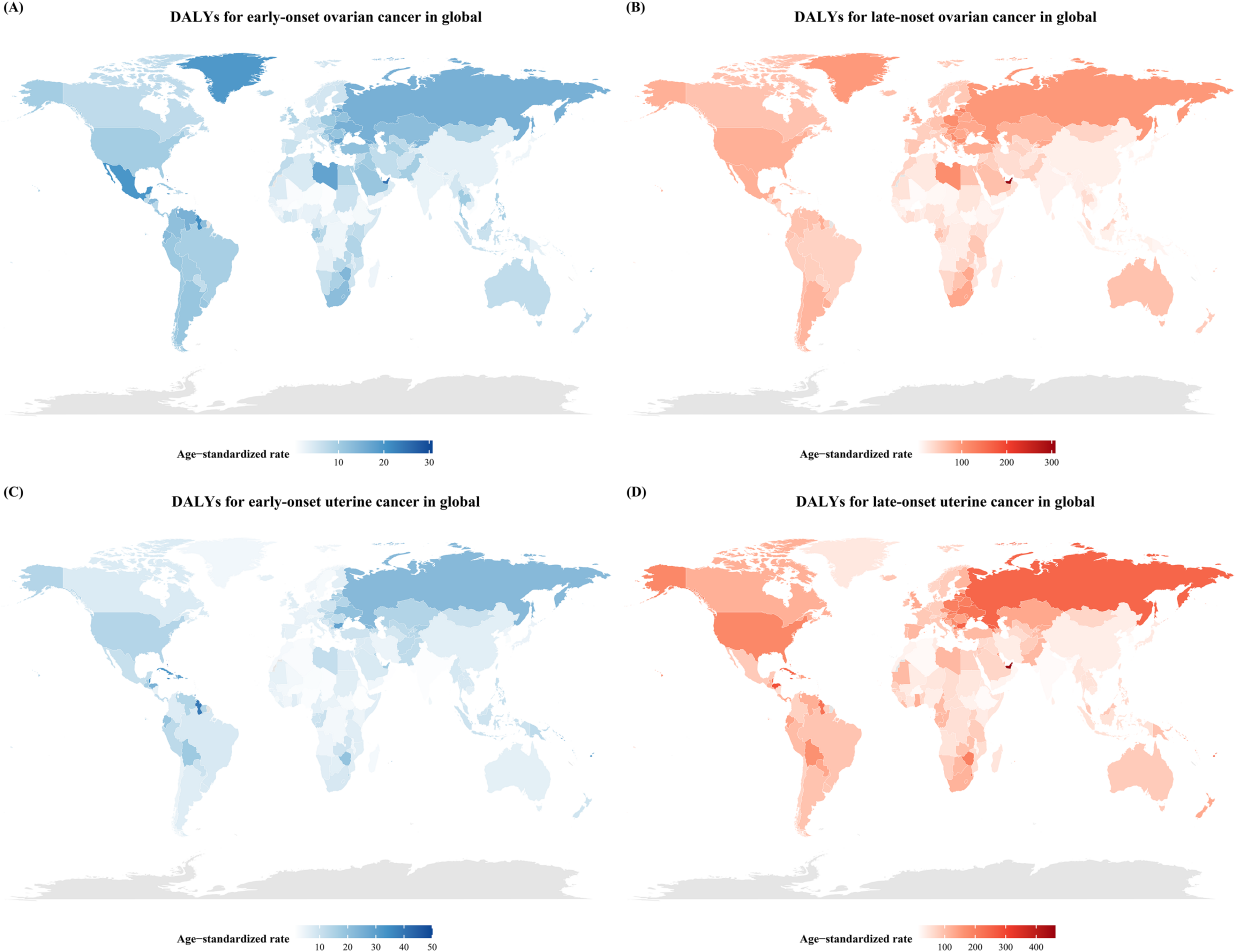
The heat map of the ASDR for ovarian cancer and uterine cancer across countries worldwide in 2021. **(A)** early-onset ovarian cancer; **(B)** late-onset ovarian cancer; **(C)** early-onset uterine cancer; **(D)** late-onset uterine cancer. Blue/red indicates early-onset/late-onset cancers, with deeper blue/red representing higher ASDR values. White areas indicate no reported cases of this cancer in the country, while gray areas indicate no collected data on this cancer in the country. A higher ASDR value indicates a greater disease burden associated with DALYs. DALYs, disability-adjusted life-years; ASDR, age-standardized rates of DALYs.

**Figure 3:**
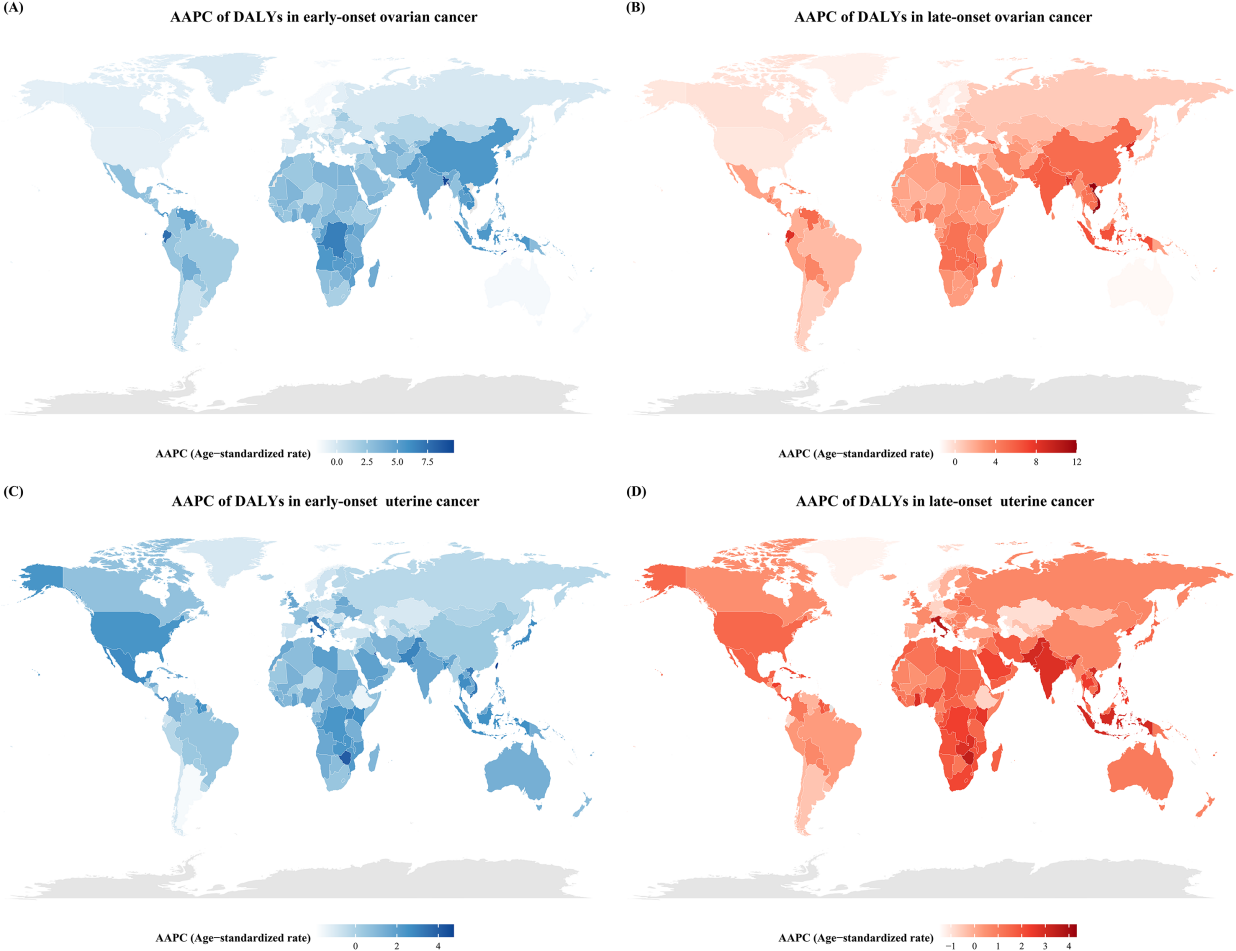
The heat map of global ASDR changes for ovarian cancer and uterine cancer from 1990 to 2021. **(A)** early-onset ovarian cancer; **(B)** late-onset ovarian cancer; **(C)** early-onset uterine cancer; **(D)** late-onset uterine cancer. Blue/red indicates early-onset/late-onset cancers, with deeper blue/red representing higher AAPC values. White areas indicate no reported cases of this cancer in the country, while gray areas indicate no collected data on this cancer in the country. A positive AAPC value indicates an overall increase in ASDR during that period, while a negative AAPC value indicates an overall decrease in ASDR during that period. DALYs, disability-adjusted life-years; ASDR, age-standardized rates of DALYs; AAPC, average annual percentage change.

**Table 2** lists the top ten countries (excluding countries with very small case numbers) with the highest ASR (ASDR, ASMR) for early-onset/late-onset ovarian cancer and uterine cancer in 2021. The highest ASDR for early-onset ovarian cancer was observed in United Arab Emirates [24.68 (95%CI: 6.44 to 51.18)] and Mexico [ASDR: 19.98 (95%CI: 5.66 to 36.41)]. The highest ASDR for late-onset ovarian cancer was found in United Arab Emirates [ASDR: 309.07 (95%CI: 79.39 to 611.25)] and Bahamas [ASDR: 134.36 (95%CI: 38.91 to 266.36)]. For uterine cancer, the countries with the highest ASDR for early-onset cases were Trinidad and Tobago [ASDR: 38.68 (95%CI: 22.97 to 58.18)] and Jamaica [ASDR: 35.6 (95%CI: 20.3 to 56.13)]. The countries with the highest ASDR for late-onset cases were United Arab Emirates [ASDR: 464.14 (95%CI: 265.58 to 764.6)] and Honduras [ASDR: 284.58 (95%CI: 138.39 to 494.86)]. The top ten countries (retained countries with very small case numbers) with the highest ASDR/ASMR for ovarian cancer and uterine cancer in 2021 are presented in **Supplementary Table 5**. **Figure 2** presents a heat map of the ASDR for ovarian cancer and uterine cancer across countries worldwide. The global heat map of the AAPC of ASDR for ovarian cancer and uterine cancer is shown in **Figure 3**. Early-onset ovarian cancer exhibited higher AAPC in Timor-Leste, Bangladesh, and Ecuador (**Figure 3A**). For late-onset ovarian cancer, high AAPC was found in Viet Nam, Bangladesh, and Democratic People’s Republic of Korea (**Figure 3B**). Early-onset uterine cancer showed higher AAPC in Zimbabwe, Kuwait, and Lesotho (**Figure 3C**). High AAPC of late-onset uterine cancer was observed in Italy, Zimbabwe, and Jamaica (**Figure 3D**).

**Table 2:** The top ten countries with the highest ASR for early-onset/late-onset ovarian cancer and uterine cancer attributable to high BMI in 2021.

Cancer	Countries	Number in 1990 (95% CI)	ASR in 1990 (95% CI)	Number in 2021 (95% CI)	ASR in 2021 (95%CI)	AAPC of ASR (95% CI)	*P*
DALYs							
Early-onset ovarian cancer	United Arab Emirates	25 (4 to 64)	12.74 (2.13 to 32.23)	448 (117 to 922)	24.68 (6.44 to 51.18)	2.277 (2.178 to 2.390)	<0.001
	Mexico	1217 (264 to 2290)	8.98 (1.99 to 16.76)	6032 (1713 to 10992)	19.98 (5.66 to 36.41)	2.651 (2.530 to 2.775)	<0.001
	Jamaica	35 (8 to 70)	9.91 (2.13 to 19.43)	112 (31 to 227)	17.99 (5.08 to 36.34)	2.250 (2.040 to 2.460)	<0.001
	Libya	30 (5 to 70)	6.27 (1 to 14.68)	315 (73 to 665)	17.49 (4.04 to 36.98)	3.359 (3.333 to 3.385)	<0.001
	Bulgaria	248 (45 to 488)	12.62 (2.24 to 24.99)	277 (63 to 554)	16.79 (3.7 to 33.85)	0.881 (0.585 to 1.108)	<0.001
	Georgia	39 (8 to 80)	3.78 (0.78 to 7.71)	127 (28 to 246)	16.09 (3.52 to 31.1)	4.955 (4.562 to 5.317)	<0.001
	Russian Federation	4451 (968 to 8201)	15.13 (3.29 to 27.8)	5554 (1379 to 10097)	15.15 (3.74 to 27.6)	-0.071 (-0.201 to 0.080)	0.315
	Ukraine	1258 (271 to 2534)	11.37 (2.44 to 22.88)	1695 (352 to 3737)	15.14 (3.13 to 33.42)	0.989 (0.859 to 1.160)	<0.001
	Venezuela (Bolivarian Republic of)	99 (19 to 199)	3.03 (0.61 to 6.05)	942 (244 to 1873)	14.92 (3.84 to 29.72)	5.200 (4.803 to 5.597)	<0.001
	El Salvador	45 (9 to 94)	5.57 (1.16 to 11.44)	202 (48 to 415)	14.09 (3.35 to 28.86)	3.132 (3.009 to 3.237)	<0.001
Late-onset ovarian cancer	United Arab Emirates	28 (4 to 73)	88.8 (14.11 to 232.2)	461 (126 to 931)	309.07 (79.39 to 611.25)	3.977 (3.750 to 4.209)	<0.001
	Bahrain	14 (3 to 30)	77.5 (14.72 to 160.7)	124 (37 to 246)	134.36 (38.91 to 266.36)	1.828 (1.728 to 1.935)	<0.001
	Latvia	399 (94 to 753)	82.61 (19.55 to 155.73)	569 (140 to 1082)	126.26 (31.13 to 239.66)	1.429 (1.199 to 1.685)	<0.001
	Serbia	938 (195 to 1898)	64.37 (13.33 to 130.9)	2082 (564 to 3912)	114.27 (30.62 to 215.67)	1.790 (1.685 to 1.903)	<0.001
	Poland	4978 (1136 to 9275)	91.1 (20.82 to 169.75)	9273 (2305 to 17136)	109.98 (27.33 to 203.27)	0.563 (0.480 to 0.644)	<0.001
	Libya	83 (16 to 176)	42.42 (8.35 to 90.04)	664 (179 to 1321)	109.7 (29.51 to 219.41)	3.118 (3.088 to 3.148)	<0.001
	Georgia	181 (41 to 360)	21.45 (4.84 to 42.82)	794 (183 to 1555)	108.78 (25.03 to 212.36)	5.565 (5.245 to 5.841)	<0.001
	Bulgaria	872 (197 to 1614)	58.54 (13.22 to 108.27)	1573 (373 to 3059)	103.29 (24.6 to 201.55)	1.970 (1.752 to 2.207)	<0.001
	Slovakia	715 (169 to 1387)	97.99 (23.1 to 190.06)	1154 (292 to 2257)	102.8 (25.77 to 201.17)	0.118 (0.051 to 0.190)	0.001
	Lithuania	466 (105 to 895)	77.72 (17.5 to 149.03)	703 (170 to 1337)	100.28 (24.22 to 191.09)	0.925 (0.619 to 1.254)	<0.001
Early-onset uterine cancer	Trinidad and Tobago	53 (34 to 72)	24.48 (16 to 33.54)	125 (74 to 187)	38.68 (22.97 to 58.18)	1.439 (1.192 to 1.677)	<0.001
	Jamaica	52 (33 to 76)	14.9 (9.43 to 21.62)	222 (126 to 350)	35.60 (20.30 to 56.13)	2.894 (2.481 to 3.249)	<0.001
	Cuba	515 (323 to 764)	22.92 (14.37 to 33.96)	820 (506 to 1243)	32.12 (19.81 to 48.79)	1.115 (0.949 to 1.275)	<0.001
	Dominican Republic	183 (95 to 315)	16.45 (8.57 to 28.2)	669 (337 to 1152)	29.19 (14.72 to 50.23)	1.876 (1.768 to 1.983)	<0.001
	Bulgaria	477 (285 to 716)	24.35 (14.52 to 36.62)	482 (308 to 704)	29.08 (18.51 to 42.78)	0.428 (-0.024 to 0.835)	0.062
	Honduras	99 (48 to 179)	15.98 (7.78 to 28.78)	474 (214 to 891)	24.58 (11.19 to 45.67)	1.441 (1.306 to 1.605)	<0.001
	Haiti	156 (61 to 300)	15.74 (6.29 to 30.06)	648 (288 to 1233)	24.35 (10.84 to 46.53)	1.514 (1.446 to 1.573)	<0.001
	Georgia	343 (224 to 474)	32.14 (21.05 to 44.25)	180 (124 to 251)	22.7 (15.55 to 31.67)	-1.458 (-1.918 to -0.976)	<0.001
	Russian Federation	7452 (5101 to 9992)	25.05 (17.19 to 33.63)	8201 (5566 to 11059)	22.29 (15.1 to 30.08)	-0.261 (-0.460 to -0.050)	0.015
	Ukraine	1412 (882 to 2021)	12.73 (7.96 to 18.22)	2266 (1134 to 3877)	20.2 (10.08 to 34.65)	1.302 (0.394 to 2.051)	0.005
Late-onset uterine cancer	United Arab Emirates	65 (27 to 127)	213.21 (89.03 to 414.76)	583 (330 to 974)	464.14 (265.58 to 764.6)	2.412 (2.157 to 2.651)	<0.001
	Honduras	323 (168 to 558)	140.68 (73.02 to 243.26)	2152 (1047 to 3740)	284.58 (138.39 to 494.86)	2.360 (2.229 to 2.470)	<0.001
	Barbados	55 (36 to 78)	165.58 (107.97 to 232.63)	182 (117 to 262)	284.37 (183.56 to 409.09)	1.840 (1.682 to 1.999)	<0.001
	Jamaica	210 (131 to 313)	106.41 (66.98 to 158.14)	958 (587 to 1453)	276.16 (169.26 to 418.6)	3.221 (2.998 to 3.428)	<0.001
	Georgia	2051 (1392 to 2794)	237.49 (161.31 to 323.4)	1950 (1285 to 2763)	255.82 (168.61 to 362.12)	0.082 (-0.274 to 0.464)	0.707
	Trinidad and Tobago	159 (103 to 224)	169.74 (109.92 to 239.05)	582 (367 to 850)	253.3 (159.5 to 370.49)	1.373 (1.167 to 1.585)	<0.001
	Bulgaria	2821 (1800 to 4083)	183.72 (116.95 to 265.65)	4267 (2748 to 6133)	251 (162.16 to 359.91)	0.853 (0.612 to 1.070)	<0.001
	Russian Federation	52089 (36384 to 68948)	197.21 (137.79 to 260.72)	81038 (56812 to 106664)	247.98 (173.99 to 326.2)	0.743 (0.603 to 0.890)	<0.001
	Bahamas	25 (16 to 36)	138.86 (89.71 to 197.39)	125 (82 to 178)	242.2 (158.44 to 343.83)	1.838 (1.647 to 2.037)	<0.001
	Cuba	1468 (917 to 2161)	132.44 (82.88 to 194.73)	5596 (3520 to 8362)	241.58 (152.37 to 361.16)	2.150 (1.956 to 2.380)	<0.001
Mortality							
Early-onset ovarian cancer	Mexico	24 (5 to 45)	0.18 (0.04 to 0.34)	122 (35 to 221)	0.4 (0.12 to 0.73)	2.681 (2.562 to 2.804)	<0.001
	Russian Federation	92 (20 to 168)	0.32 (0.07 to 0.58)	115 (29 to 210)	0.31 (0.08 to 0.57)	-0.123 (-0.254 to 0.020)	0.091
	Ukraine	26 (6 to 52)	0.24 (0.05 to 0.47)	35 (7 to 78)	0.31 (0.06 to 0.69)	0.952 (0.820 to 1.125)	<0.001
	South Africa	9 (2 to 19)	0.15 (0.03 to 0.32)	32 (8 to 59)	0.26 (0.07 to 0.47)	1.779 (1.663 to 1.919)	<0.001
	Colombia	6 (1 to 13)	0.12 (0.02 to 0.24)	27 (6 to 53)	0.25 (0.06 to 0.49)	2.432 (2.335 to 2.521)	<0.001
	Turkey	19 (4 to 45)	0.21 (0.04 to 0.49)	50 (12 to 97)	0.25 (0.06 to 0.48)	0.599 (0.549 to 0.658)	<0.001
	Argentina	13 (2 to 28)	0.21 (0.04 to 0.43)	24 (6 to 45)	0.23 (0.05 to 0.44)	0.312 (0.234 to 0.393)	<0.001
	Saudi Arabia	2 (0 to 4)	0.09 (0.02 to 0.21)	20 (5 to 43)	0.23 (0.06 to 0.49)	2.982 (2.946 to 3.019)	<0.001
	Poland	21 (3 to 44)	0.28 (0.04 to 0.58)	22 (5 to 41)	0.22 (0.05 to 0.43)	-0.859 (-1.026 to -0.69)	<0.001
	Pakistan	7 (0 to 18)	0.04 (0 to 0.12)	84 (14 to 187)	0.2 (0.03 to 0.44)	5.021 (4.973 to 5.064)	<0.001
Late-onset ovarian cancer	Latvia	15 (4 to 29)	2.98 (0.7 to 5.64)	25 (6 to 47)	4.71 (1.16 to 8.97)	1.561 (1.403 to 1.735)	<0.001
	Poland	193 (44 to 361)	3.36 (0.77 to 6.28)	416 (102 to 771)	4.4 (1.09 to 8.14)	0.811 (0.732 to 0.888)	<0.001
	Serbia	34 (7 to 69)	2.41 (0.49 to 4.93)	87 (23 to 164)	4.35 (1.17 to 8.21)	1.906 (1.798 to 2.033)	<0.001
	Libya	3 (1 to 6)	1.54 (0.29 to 3.31)	23 (6 to 47)	4.11 (1.09 to 8.31)	3.206 (3.171 to 3.242)	<0.001
	Georgia	6 (1 to 13)	0.74 (0.17 to 1.48)	32 (7 to 62)	4.07 (0.93 to 7.98)	5.819 (5.437 to 6.136)	<0.001
	Lithuania	18 (4 to 35)	2.9 (0.65 to 5.55)	32 (8 to 60)	3.95 (0.96 to 7.48)	1.102 (0.816 to 1.404)	<0.001
	Slovakia	28 (7 to 54)	3.63 (0.85 to 7.05)	48 (12 to 95)	3.95 (1 to 7.76)	0.237 (0.170 to 0.306)	<0.001
	Bulgaria	32 (7 to 60)	2.13 (0.48 to 3.97)	66 (15 to 128)	3.79 (0.9 to 7.37)	2.076 (1.906 to 2.303)	<0.001
	Hungary	65 (15 to 120)	3.33 (0.77 to 6.12)	92 (23 to 169)	3.69 (0.96 to 6.77)	0.288 (0.131 to 0.438)	<0.001
	Russian Federation	752 (181 to 1392)	2.85 (0.69 to 5.26)	1215 (312 to 2140)	3.68 (0.95 to 6.46)	0.722 (0.577 to 0.932)	<0.001
Early-onset uterine cancer	Russian Federation	144 (99 to 193)	0.49 (0.34 to 0.66)	152 (104 to 205)	0.41 (0.28 to 0.56)	-0.449 (-0.648 to -0.241)	<0.001
	Ukraine	29 (18 to 41)	0.26 (0.16 to 0.37)	45 (23 to 78)	0.4 (0.2 to 0.69)	1.218 (0.308 to 1.967)	0.011
	United States of America	71 (50 to 94)	0.13 (0.09 to 0.17)	172 (126 to 216)	0.25 (0.18 to 0.31)	2.120 (1.949 to 2.352)	<0.001
	Pakistan	15 (9 to 26)	0.1 (0.06 to 0.17)	101 (53 to 169)	0.24 (0.13 to 0.4)	2.857 (2.824 to 2.888)	<0.001
	Mexico	15 (11 to 20)	0.12 (0.08 to 0.16)	66 (44 to 90)	0.22 (0.15 to 0.3)	2.667 (2.412 to 2.930)	<0.001
	Philippines	12 (6 to 18)	0.12 (0.07 to 0.19)	45 (25 to 70)	0.21 (0.12 to 0.32)	1.805 (1.761 to 1.848)	<0.001
	Indonesia	28 (13 to 47)	0.09 (0.04 to 0.15)	131 (63 to 216)	0.2 (0.09 to 0.32)	2.531 (2.510 to 2.549)	<0.001
	Turkey	24 (11 to 42)	0.26 (0.12 to 0.46)	37 (20 to 61)	0.18 (0.1 to 0.3)	-1.099 (-1.210 to -0.994)	<0.001
	Myanmar	10 (4 to 22)	0.17 (0.06 to 0.34)	21 (10 to 40)	0.17 (0.08 to 0.32)	0.030 (0.006 to 0.056)	0.016
	Brazil	33 (22 to 46)	0.13 (0.09 to 0.19)	85 (59 to 113)	0.16 (0.11 to 0.21)	0.410 (0.200 to 0.725)	0.016
Late-onset uterine cancer	United Arab Emirates	2 (1 to 4)	7.84 (3.36 to 15.23)	21 (12 to 34)	22.32 (12.7 to 36.75)	3.317 (3.011 to 3.618)	<0.001
	Honduras	12 (6 to 21)	5.34 (2.76 to 9.34)	82 (39 to 143)	11.01 (5.3 to 19.24)	2.400 (2.251 to 2.557)	<0.001
	Jamaica	9 (5 to 13)	4.07 (2.56 to 6.12)	39 (24 to 59)	10.76 (6.56 to 16.27)	3.301 (3.005 to 3.617)	<0.001
	Georgia	77 (52 to 105)	8.74 (5.89 to 11.94)	80 (53 to 113)	9.75 (6.46 to 13.79)	0.218 (-0.153 to 0.616)	0.266
	Trinidad and Tobago	6 (4 to 9)	6.48 (4.15 to 9.21)	23 (15 to 34)	9.71 (6.1 to 14.18)	1.362 (1.080 to 1.601)	<0.001
	Bulgaria	107 (68 to 155)	6.91 (4.39 to 10.02)	184 (117 to 265)	9.48 (6.09 to 13.65)	0.904 (0.710 to 1.076)	<0.001
	Cuba	57 (36 to 85)	4.97 (3.1 to 7.35)	233 (146 to 350)	9.45 (5.92 to 14.15)	2.289 (2.108 to 2.501)	<0.001
	Russian Federation	1938 (1352 to 2577)	7.07 (4.94 to 9.39)	3177 (2226 to 4186)	8.98 (6.3 to 11.82)	0.741 (0.574 to 0.907)	<0.001
	Poland	360 (248 to 485)	5.96 (4.11 to 8.04)	903 (613 to 1232)	8.43 (5.76 to 11.48)	1.083 (0.974 to 1.174)	<0.001
	Slovakia	81 (48 to 124)	10.01 (5.91 to 15.4)	112 (64 to 178)	8.42 (4.8 to 13.51)	-0.683 (-0.750 to -0.615)	<0.001

DALYs, disability-adjusted life-years; BMI, body mass index; ASR, age-standardized rate; AAPC, average annual percentage change; CI, confidence interval.

### Future trends in mortality and DALYs of ovarian cancer and uterine cancer attributable to high BMI

**Figure 4:**
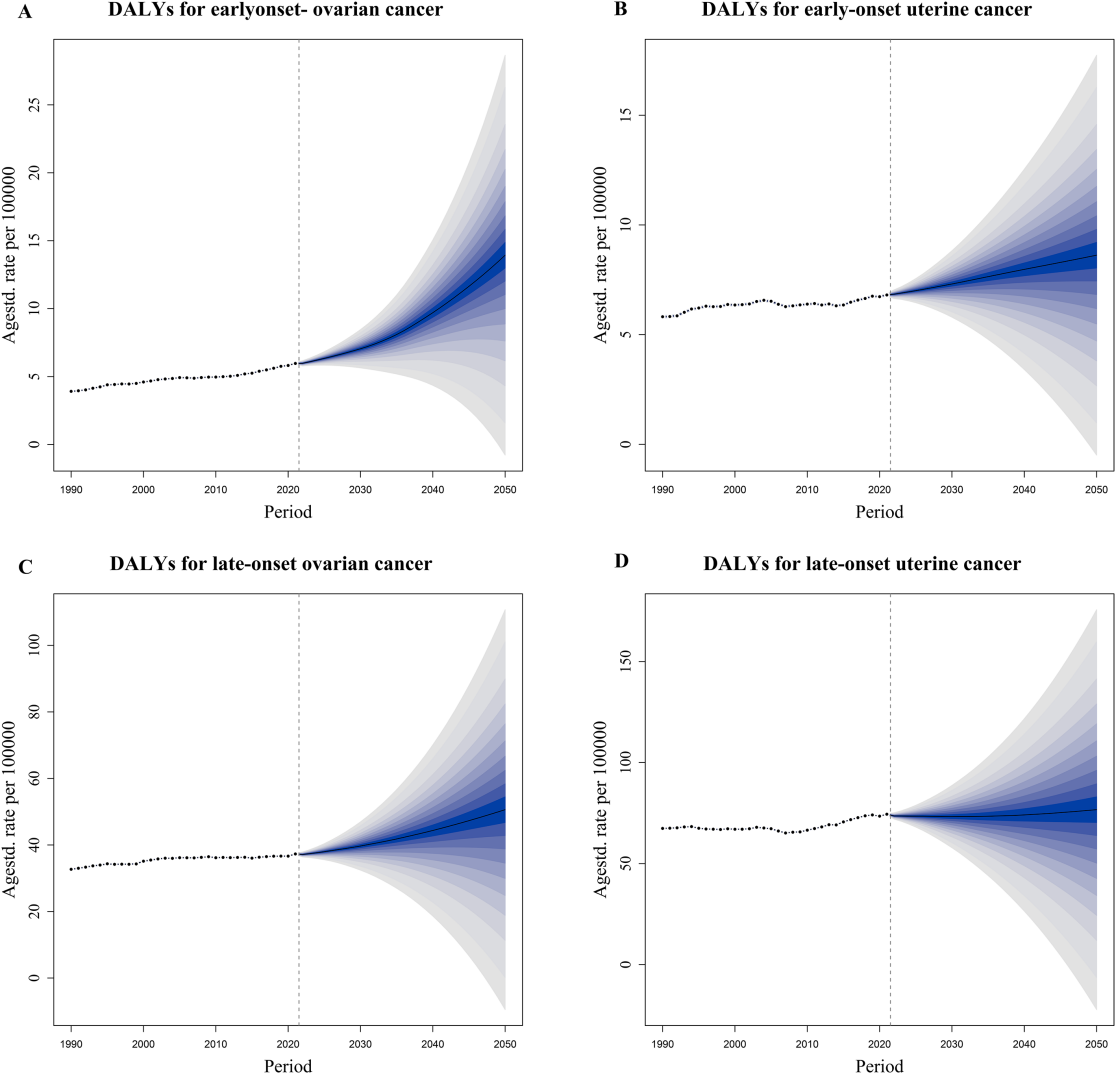
The predictive trends of ASDR in ovarian cancer and uterine cancer attributable to high BMI from 2022 to 2050. **(A)** early-onset ovarian cancer; **(B)** early-onset uterine cancer; **(C)** late-onset ovarian cancer; **(D)** late-onset uterine cancer. A higher ASDR value indicates a greater disease burden associated with DALYs. DALYs, disability-adjusted life-years; ASDR, age-standardized rates of DALYs; BMI, body mass index.

From 2022 to 2050, the ASDR and ASMR for both early-onset and late-onset ovarian cancer are projected to show a significant upward trend, but the rate of ASDR and ASMR in late-onset ovarian cancer is relatively slower than that in early-onset ovarian cancer. The ASDR and ASMR of early-onset and late-onset uterine cancer are expected to rise from 2022 to 2050, but the upward trend of ASDR and ASMR in late-onset uterine cancer is not pronounced. The trends in ASDR (**Figure 4**) and ASMR (**Supplementary Figure 1**) are similar.

## Discussion

The current study analyzed trends in mortality and DALYs for early-onset and late-onset ovarian cancer and uterine cancer attributable to high BMI from 1990 to 2021. The mortality and DALYs for early-onset and late-onset ovarian cancer and uterine cancer all showed an increasing trend from 1990 to 2021. Among regions of different economic levels, the mortality and DALYs of early-onset and late-onset ovarian cancer exhibited a declining trend in high-SDI regions, while they showed an increasing trend in other SDI regions. For uterine cancer, the mortality and DALYs of early-onset and late-onset cancer presented a downward trend in high-middle-SDI regions. Furthermore, the ASDR and ASMR for early-onset and late-onset ovarian cancer and uterine cancer are projected to rise from 2022 to 2050, but the growth rate of ASDR and ASMR in late-onset cancers is relatively lower than that in early-onset cancers.

Differences between early-onset and later-onset cancers exist in epidemiology, clinical characteristics, pathological, and molecular profiles (16, 30). The changes in the interaction between genes and the environment may be a primary cause of early-onset cancer (16). Early-onset cancers may carry a higher risk of secondary cancers and cardiovascular diseases compared to late-onset cancers (31). Furthermore, high BMI is an attributable risk factor for the disease burden of ovarian cancer and uterine cancer (6). The mechanisms by which high BMI affects ovarian cancer and uterine cancer are different (9, 11), but hormonal imbalance plays a significant role in the development of both types of cancer. Previous studies have found that the incidence of ovarian cancer and uterine cancer is on the rise among women worldwide (4, 6). The global increase in obesity rates may be an important factor contributing to increased incidence rates, as obesity alters inflammatory, metabolic, and hormonal pathways (32, 33). The present study investigated the trends in mortality and DALYs for early-onset and late-onset ovarian cancer and uterine cancer attributable to high BMI from 1990 to 2021. The results revealed that the DALYs number of late-onset ovarian cancer was much higher than that of those with early-onset cancer, and the ASDR of late-onset patients was also higher (37.30 vs. 5.97). This may be related to the peak incidence of ovarian cancer occurring between the ages of 55 and 59 (34). Similar results were found in late-onset and early-onset uterine cancer. The impact of high BMI on ovarian cancer risk may be related to the menopausal status. Overweight and obesity are linked to an elevated ovarian cancer risk in premenopausal women, whereas no significant association has been observed in postmenopausal women (35). The association between high BMI and the risk of ovarian cancer is also influenced by histological subtype. The relationship between high BMI and an increased risk is most pronounced for borderline serous tumors, invasive endometrioid carcinomas, and invasive mucinous tumors, but no significant association was observed with invasive serous carcinomas (36). Furthermore, our results indicated that the mortality and DALYs for both early-onset and late-onset ovarian cancer and uterine cancer with high BMI exhibited an upward trend from 1990 to 2021. However, several studies demonstrated that the mortality of overall ovarian cancer is on a downward trend (37, 38). This decline may be related to the use of oral contraceptives, reduced use of menopausal hormones, and improvements in diagnosis, management, and treatment (37). Reproductive factors such as parity, tubal ligation, age at menopause, and oral contraceptive use have also been reported to influence the risk of ovarian cancer (39, 40). These findings suggest that within the global trend of declining ovarian cancer mortality, particular attention may be needed for patients with high BMI, as mortality and DALYs in this population are on the rise, and this trend is projected to persist through 2050.

Socioeconomic status and lifestyle patterns may influence cancer development (41, 42). When the social economy rapidly develops to a medium or higher SDI level, improved unhealthy lifestyles and screening may lead to an increased cancer incidence (43). Conversely, in regions with higher SDI levels, healthier lifestyles and effective preventive measures may reduce cancer occurrence (43). Our results demonstrated that the mortality and DALYs of early-onset and late-onset ovarian cancer decreased in high-SDI regions, while increasing in low-middle SDI, low SDI, middle SDI, and high-middle SDI regions. For uterine cancer, the mortality and DALYs of early-onset and late-onset cases decreased in high-middle-SDI regions. Previous studies have also indicated that the mortality of ovarian cancer decreased in high-SDI regions, while the DALYs of uterine cancer increased in high-SDI regions (6). In regions with high economic levels, advances in cancer screening and treatment have significantly improved patient survival (3, 44). However, the increase in mortality and DALYs for uterine cancer in high-SDI regions may be correlated with high BMI. High BMI is a key risk factor for death from uterine cancer, and overweight or obese women are more susceptible to developing uterine cancer (45, 46). Therefore, physical exercise, healthy weight, active lifestyles, and awareness of women’s cancer prevention may help reduce population-level burden attributable to high BMI (47, 48).

This study analyzed the global and regional trends in the disease burden of early-onset/late-onset ovarian cancer and uterine cancer attributable to high BMI, and projected future trends. The representative data from the GBD study in multiple regions worldwide ensure the accuracy of mortality and DALYs estimates for ovarian cancer and uterine cancer. However, several limitations of this study should be noted. First, although GBD data are comprehensive and undergo quality control, they may underestimate the cancer burden in underdeveloped regions due to incomplete cancer registries and limited public database resources. Second, since the analyzed data span from 1990 to 2021, the COVID-19 pandemic may introduce significant uncertainty into mortality estimates for all diseases. Third, the GBD did not collect information on histological subtypes and ethnicity, limiting disease burden analyses based on cancer subtypes and ethnicity. Fourth, we adopted a 50-year age cutoff, a common cutoff in epidemiological studies, to distinguish between early-onset and late-onset cancers. However, for gynecological cancers, menopausal status may be a more biologically significant classification criterion than age, particularly for ovarian cancer. Due to limitations in the GBD database, data on menopausal status were not available.

## Conclusions

The mortality and DALYs of early-onset and late-onset ovarian cancer and uterine cancer attributable to high BMI all showed an upward trend from 1990 to 2021. The disease burden for late-onset ovarian cancer and uterine cancer was significantly higher than that for their early-onset cases. Furthermore, the mortality and DALYs for ovarian cancer and uterine cancer are projected to continue rising from 2022 to 2050. Therefore, enhancing BMI management, facilitating early detection, and ensuring equitable access to healthcare are essential strategies for mitigating this trend.

## Data Availability

The datasets presented in this study can be found in online repositories. The names of the repository/repositories and accession number(s) can be found below: All data were derived from the Global Burden of Disease study 2021, https://ghdx.healthdata.org/gbd-2021.
