# Supplementary material for: Global, regional, and national burden of ovarian cancer and uterine cancer attributable to high BMI, 1990-2021: analysis of data from the global burden of disease study 2021
Source: Front Oncol. 2026 May 28;16:1821189. doi: 10.3389/fonc.2026.1821189 (PMC13253308; doi:10.3389/fonc.2026.1821189)
Supplement: Supplementary file 1 [file DataSheet1.docx]

**Supplement Table 1**. Mortality and DALYs of early-onset ovarian cancer attributable to high BMI from 1990 to 2021 in 21 GBD regions.

| GBD regions | Number in 1990 (95% CI) | ASR in 1990 (95% CI) | Number in 2021 (95% CI) | ASR in 2021 (95% CI) | AAPC of ASR (95%CI) | *P* |
| --- | --- | --- | --- | --- | --- | --- |
| **DALYs** |  |  |  |  |  |  |
| Andean Latin America | 224 (32 to 495) | 3.74 (0.57 to 8.22) | 1611 (391 to 3157) | 11.35 (2.76 to 22.23) | 3.643 (3.507 to 3.780) | <0.001 |
| Australasia | 469 (96 to 935) | 10.53 (2.16 to 20.96) | 479 (116 to 862) | 7 (1.69 to 12.61) | -1.290 (-1.448 to -1.129) | <0.001 |
| Caribbean | 309 (62 to 578) | 4.85 (0.99 to 9.07) | 1095 (252 to 2061) | 10.74 (2.47 to 20.21) | 2.685 (2.536 to 2.868) | <0.001 |
| Central Asia | 535 (99 to 1031) | 5.62 (1.11 to 10.73) | 1902 (405 to 3560) | 9.28 (1.98 to 17.35) | 1.652 (1.521 to 1.768) | <0.001 |
| Central Europe | 3043 (581 to 5915) | 11.56 (2.21 to 22.44) | 3144 (725 to 5783) | 11.03 (2.52 to 20.33) | -0.209 (-0.315 to -0.120) | <0.001 |
| Central Latin America | 1787 (371 to 3349) | 6.83 (1.46 to 12.73) | 9348 (2621 to 17046) | 16.28 (4.57 to 29.69) | 2.929 (2.846 to 3.013) | <0.001 |
| Central Sub-Saharan Africa | 36 (-8 to 108) | 0.51 (-0.09 to 1.48) | 577 (81 to 1273) | 2.82 (0.41 to 6.2) | 5.655 (5.619 to 5.694) | <0.001 |
| East Asia | 1201 (-1073 to 3954) | 0.57 (-0.45 to 1.82) | 10913 (1885 to 22823) | 3.1 (0.52 to 6.51) | 5.550 (5.466 to 5.652) | <0.001 |
| Eastern Europe | 6137 (1309 to 11240) | 13.61 (2.91 to 24.91) | 7797 (1838 to 14174) | 14.86 (3.48 to 27.05) | 0.264 (0.168 to 0.391) | <0.001 |
| Eastern Sub-Saharan Africa | 333 (-18 to 790) | 1.39 (-0.03 to 3.23) | 3113 (477 to 6340) | 4.72 (0.75 to 9.57) | 4.038 (4.022 to 4.058) | <0.001 |
| High-income Asia Pacific | 572 (-204 to 1521) | 1.36 (-0.52 to 3.65) | 944 (47 to 2109) | 2.1 (0.05 to 4.77) | 1.418 (1.325 to 1.556) | <0.001 |
| High-income North America | 7245 (1753 to 13165) | 11.54 (2.79 to 20.94) | 7493 (2017 to 12866) | 9.68 (2.6 to 16.63) | -0.580 (-0.645 to -0.521) | <0.001 |
| North Africa and Middle East | 2181 (406 to 4679) | 4.54 (0.86 to 9.68) | 11324 (3073 to 20145) | 8.65 (2.35 to 15.39) | 2.129 (2.110 to 2.150) | <0.001 |
| Oceania | 24 (5 to 51) | 2.53 (0.49 to 5.28) | 112 (23 to 239) | 4.38 (0.93 to 9.35) | 1.889 (1.829 to 1.955) | <0.001 |
| South Asia | 1335 (-103 to 3138) | 0.82 (-0.04 to 1.89) | 13483 (2311 to 26406) | 3.56 (0.62 to 6.96) | 4.872 (4.825 to 4.926) | <0.001 |
| Southeast Asia | 1223 (-29 to 2849) | 1.63 (0 to 3.73) | 9753 (1805 to 19029) | 6.12 (1.13 to 11.96) | 4.381 (4.365 to 4.399) | <0.001 |
| Southern Latin America | 858 (166 to 1738) | 8.92 (1.73 to 18.05) | 1707 (422 to 3221) | 11.05 (2.73 to 20.84) | 0.732 (0.590 to 0.865) | <0.001 |
| Southern Sub-Saharan Africa | 552 (118 to 1153) | 6.63 (1.45 to 13.72) | 2146 (526 to 3988) | 12.72 (3.12 to 23.63) | 2.116 (2.012 to 2.257) | <0.001 |
| Tropical Latin America | 1431 (230 to 2871) | 5.36 (0.91 to 10.63) | 5172 (1222 to 9458) | 9.47 (2.23 to 17.33) | 1.786 (1.616 to 2.005) | <0.001 |
| Western Europe | 5879 (1030 to 11217) | 7.08 (1.24 to 13.51) | 5624 (1293 to 10321) | 5.82 (1.34 to 10.7) | -0.640 (-0.717 to -0.560) | <0.001 |
| Western Sub-Saharan Africa | 269 (33 to 543) | 1.09 (0.15 to 2.18) | 2179 (447 to 4287) | 2.93 (0.61 to 5.74) | 3.165 (3.128 to 3.198) | <0.001 |
| **Mortality** |  |  |  |  |  |  |
| Andean Latin America | 4 (1 to 10) | 0.08 (0.01 to 0.17) | 32 (8 to 63) | 0.23 (0.06 to 0.44) | 3.565 (3.434 to 3.698) | <0.001 |
| Australasia | 10 (2 to 19) | 0.21 (0.04 to 0.42) | 10 (2 to 17) | 0.14 (0.03 to 0.25) | -1.350 (-1.518 to -1.180) | <0.001 |
| Caribbean | 6 (1 to 12) | 0.1 (0.02 to 0.18) | 22 (5 to 42) | 0.22 (0.05 to 0.41) | 2.670 (2.521 to 2.856) | <0.001 |
| Central Asia | 11 (2 to 21) | 0.12 (0.02 to 0.23) | 39 (8 to 73) | 0.19 (0.04 to 0.36) | 1.587 (1.449 to 1.702) | <0.001 |
| Central Europe | 64 (13 to 123) | 0.24 (0.05 to 0.47) | 66 (15 to 120) | 0.23 (0.05 to 0.42) | -0.275 (-0.370 to -0.182) | <0.001 |
| Central Latin America | 35 (7 to 66) | 0.14 (0.03 to 0.26) | 188 (53 to 342) | 0.33 (0.09 to 0.6) | 2.901 (2.822 to 2.982) | <0.001 |
| Central Sub-Saharan Africa | 1 (0 to 2) | 0.01 (0 to 0.03) | 12 (2 to 26) | 0.06 (0.01 to 0.13) | 5.573 (5.536 to 5.613) | <0.001 |
| East Asia | 25 (-20 to 80) | 0.01 (-0.01 to 0.04) | 226 (40 to 471) | 0.06 (0.01 to 0.13) | 5.446 (5.361 to 5.552) | <0.001 |
| Eastern Europe | 127 (27 to 232) | 0.29 (0.06 to 0.52) | 162 (38 to 294) | 0.31 (0.07 to 0.56) | 0.220 (0.120 to 0.352) | <0.001 |
| Eastern Sub-Saharan Africa | 7 (0 to 16) | 0.03 (0 to 0.07) | 62 (10 to 125) | 0.1 (0.02 to 0.19) | 3.950 (3.933 to 3.972) | <0.001 |
| High-income Asia Pacific | 12 (-4 to 32) | 0.03 (-0.01 to 0.08) | 20 (1 to 44) | 0.04 (0 to 0.1) | 1.308 (1.219 to 1.432) | <0.001 |
| High-income North America | 146 (35 to 265) | 0.23 (0.06 to 0.42) | 150 (40 to 258) | 0.19 (0.05 to 0.33) | -0.644 (-0.707 to -0.588) | <0.001 |
| North Africa and Middle East | 44 (8 to 94) | 0.09 (0.02 to 0.2) | 227 (62 to 404) | 0.17 (0.05 to 0.31) | 2.066 (2.048 to 2.087) | <0.001 |
| Oceania | 0 (0 to 1) | 0.05 (0.01 to 0.11) | 2 (0 to 5) | 0.09 (0.02 to 0.19) | 1.875 (1.805 to 1.953) | <0.001 |
| South Asia | 28 (-1 to 65) | 0.02 (0 to 0.04) | 273 (48 to 532) | 0.07 (0.01 to 0.14) | 4.750 (4.700 to 4.805) | <0.001 |
| Southeast Asia | 25 (0 to 57) | 0.03 (0 to 0.08) | 194 (36 to 376) | 0.12 (0.02 to 0.24) | 4.263 (4.241 to 4.282) | <0.001 |
| Southern Latin America | 18 (3 to 35) | 0.18 (0.04 to 0.37) | 35 (9 to 65) | 0.22 (0.06 to 0.42) | 0.629 (0.498 to 0.759) | <0.001 |
| Southern Sub-Saharan Africa | 11 (2 to 22) | 0.13 (0.03 to 0.27) | 43 (10 to 80) | 0.26 (0.06 to 0.48) | 2.195 (2.091 to 2.311) | <0.001 |
| Tropical Latin America | 29 (5 to 57) | 0.11 (0.02 to 0.22) | 103 (24 to 189) | 0.19 (0.04 to 0.34) | 1.751 (1.529 to 1.945) | <0.001 |
| Western Europe | 122 (22 to 231) | 0.15 (0.03 to 0.28) | 115 (26 to 210) | 0.12 (0.03 to 0.21) | -0.728 (-0.805 to -0.644) | <0.001 |
| Western Sub-Saharan Africa | 5 (1 to 11) | 0.02 (0 to 0.04) | 43 (9 to 85) | 0.06 (0.01 to 0.12) | 3.099 (3.062 to 3.132) | <0.001 |

Note: DALYs, disability-adjusted life-years; BMI, body mass index; ASR, age-standardized rate; AAPC, average annual percentage change; CI, confidence interval.

**Supplement Table 2**. Mortality and DALYs of late-onset ovarian cancer attributable to high BMI in 21 GBD regions, 1900-2021.

| GBD regions | Number in 1990 (95% CI) | ASR in 1990 (95% CI) | Number in 2021 (95% CI) | ASR in 2021 (95% CI) | AAPC of ASR (95% CI) | *P* |
| --- | --- | --- | --- | --- | --- | --- |
| **DALYs** |  |  |  |  |  |  |
| Andean Latin America | 396 (67 to 842) | 17.23 (2.89 to 36.65) | 3408 (841 to 6609) | 50.79 (12.56 to 98.46) | 3.567 (3.466 to 3.663) | <0.001 |
| Australasia | 2347 (488 to 4469) | 89.37 (18.75 to 169.98) | 3456 (884 to 6431) | 57.99 (14.97 to 107.25) | -1.270 (-1.522 to -1.031) | <0.001 |
| Caribbean | 643 (130 to 1222) | 22.51 (4.59 to 42.77) | 2842 (709 to 5293) | 45.76 (11.47 to 85.17) | 2.364 (2.282 to 2.440) | <0.001 |
| Central Asia | 1987 (448 to 3669) | 31.83 (7.16 to 58.8) | 6035 (1507 to 10960) | 55.83 (13.91 to 101.57) | 1.822 (1.740 to 1.919) | <0.001 |
| Central Europe | 13830 (3234 to 25272) | 73.78 (17.23 to 134.78) | 24569 (6460 to 44711) | 97.25 (25.48 to 177.07) | 0.929 (0.874 to 0.983) | <0.001 |
| Central Latin America | 2963 (655 to 5531) | 31.19 (6.88 to 58.26) | 19930 (5410 to 36085) | 66.27 (18 to 119.93) | 2.440 (2.370 to 2.524) | <0.001 |
| Central Sub-Saharan Africa | 159 (4 to 392) | 5.33 (0.07 to 13.31) | 1471 (266 to 3127) | 20.54 (3.65 to 43.78) | 4.456 (4.440 to 4.474) | <0.001 |
| East Asia | 3530 (-1261 to 9376) | 3.48 (-1.26 to 9.28) | 44076 (8725 to 91352) | 16.81 (3.33 to 34.82) | 5.223 (5.176 to 5.273) | <0.001 |
| Eastern Europe | 29096 (7073 to 52585) | 74.24 (18.06 to 133.95) | 42259 (11004 to 74106) | 94.99 (24.72 to 166.35) | 0.718 (0.623 to 0.822) | <0.001 |
| Eastern Sub-Saharan Africa | 800 (20 to 1783) | 9.06 (0.11 to 20.32) | 5640 (973 to 11364) | 27.73 (4.71 to 55.93) | 3.676 (3.665 to 3.689) | <0.001 |
| High-income Asia Pacific | 1978 (-89 to 4368) | 8.13 (-0.33 to 17.95) | 5173 (564 to 10542) | 12.27 (1.45 to 24.91) | 1.355 (1.304 to 1.414) | <0.001 |
| High-income North America | 34101 (8177 to 62198) | 85.89 (20.73 to 155.82) | 56697 (15630 to 98813) | 75.08 (20.88 to 130.21) | -0.533 (-0.598 to -0.475) | <0.001 |
| North Africa and Middle East | 4813 (1049 to 9879) | 25.14 (5.45 to 51.63) | 26236 (7617 to 46113) | 51.09 (14.78 to 89.94) | 2.299 (2.278 to 2.323) | <0.001 |
| Oceania | 38 (8 to 78) | 11.15 (2.18 to 22.98) | 171 (43 to 343) | 19.41 (4.8 to 38.9) | 1.828 (1.768 to 1.878) | <0.001 |
| South Asia | 1985 (-437 to 5155) | 3.06 (-0.73 to 8) | 29132 (4915 to 57289) | 17.14 (2.89 to 33.73) | 5.771 (5.726 to 5.812) | <0.001 |
| Southeast Asia | 1535 (-96 to 3536) | 4.79 (-0.36 to 11.09) | 16887 (3099 to 32397) | 20.32 (3.7 to 39.03) | 4.773 (4.748 to 4.796) | <0.001 |
| Southern Latin America | 3383 (759 to 6498) | 60.9 (13.69 to 116.56) | 6919 (1781 to 12461) | 69.06 (17.92 to 123.97) | 0.485 (0.366 to 0.604) | <0.001 |
| Southern Sub-Saharan Africa | 1222 (302 to 2335) | 36.44 (8.99 to 69.71) | 6258 (1690 to 11158) | 83.15 (22.46 to 148.35) | 2.720 (2.659 to 2.781) | <0.001 |
| Tropical Latin America | 3195 (661 to 6196) | 29.46 (6.08 to 57.19) | 13860 (3283 to 25352) | 44.56 (10.57 to 81.39) | 1.320 (1.257 to 1.397) | <0.001 |
| Western Europe | 44467 (9089 to 83897) | 65.66 (13.47 to 123.9) | 56757 (13148 to 105020) | 55.86 (12.98 to 102.99) | -0.545 (-0.601 to -0.495) | <0.001 |
| Western Sub-Saharan Africa | 762 (119 to 1512) | 8.1 (1.26 to 16.1) | 5557 (1196 to 10840) | 22.83 (4.9 to 44.57) | 3.381 (3.340 to 3.422) | <0.001 |
| **Mortality** |  |  |  |  |  |  |
| Andean Latin America | 14 (2 to 30) | 0.6 (0.09 to 1.31) | 126 (31 to 246) | 1.85 (0.45 to 3.6) | 3.673 (3.577 to 3.765) | <0.001 |
| Australasia | 97 (20 to 186) | 3.42 (0.7 to 6.52) | 163 (41 to 308) | 2.42 (0.62 to 4.53) | -1.008 (-1.284 to -0.750) | <0.001 |
| Caribbean | 24 (5 to 45) | 0.82 (0.16 to 1.55) | 108 (26 to 202) | 1.68 (0.41 to 3.13) | 2.425 (2.336 to 2.509) | <0.001 |
| Central Asia | 71 (16 to 130) | 1.13 (0.25 to 2.08) | 216 (54 to 394) | 2.04 (0.51 to 3.74) | 1.940 (1.855 to 2.044) | <0.001 |
| Central Europe | 531 (124 to 970) | 2.74 (0.64 to 5) | 1072 (279 to 1950) | 3.79 (0.99 to 6.89) | 1.045 (0.993 to 1.095) | <0.001 |
| Central Latin America | 109 (24 to 206) | 1.17 (0.25 to 2.2) | 736 (198 to 1341) | 2.42 (0.65 to 4.41) | 2.343 (2.271 to 2.445) | <0.001 |
| Central Sub-Saharan Africa | 5 (0 to 13) | 0.19 (0 to 0.48) | 50 (9 to 106) | 0.73 (0.12 to 1.57) | 4.498 (4.483 to 4.513) | <0.001 |
| East Asia | 120 (-48 to 321) | 0.12 (-0.05 to 0.32) | 1588 (307 to 3285) | 0.6 (0.12 to 1.24) | 5.381 (5.330 to 5.441) | <0.001 |
| Eastern Europe | 1055 (255 to 1910) | 2.6 (0.63 to 4.71) | 1670 (432 to 2942) | 3.49 (0.9 to 6.13) | 0.866 (0.763 to 1.003) | <0.001 |
| Eastern Sub-Saharan Africa | 26 (0 to 59) | 0.31 (-0.01 to 0.7) | 191 (31 to 386) | 0.97 (0.16 to 1.97) | 3.812 (3.799 to 3.830) | <0.001 |
| High-income Asia Pacific | 72 (-5 to 161) | 0.29 (-0.02 to 0.64) | 233 (22 to 479) | 0.45 (0.05 to 0.92) | 1.475 (1.418 to 1.541) | <0.001 |
| High-income North America | 1458 (346 to 2683) | 3.3 (0.79 to 6.03) | 2551 (685 to 4504) | 3.06 (0.83 to 5.35) | -0.310 (-0.365 to -0.253) | <0.001 |
| North Africa and Middle East | 171 (37 to 351) | 0.93 (0.2 to 1.91) | 973 (278 to 1722) | 1.96 (0.56 to 3.48) | 2.423 (2.393 to 2.459) | <0.001 |
| Oceania | 1 (0 to 3) | 0.39 (0.07 to 0.81) | 6 (1 to 11) | 0.68 (0.16 to 1.36) | 1.872 (1.809 to 1.923) | <0.001 |
| South Asia | 65 (-17 to 172) | 0.1 (-0.03 to 0.28) | 1030 (166 to 2049) | 0.61 (0.1 to 1.22) | 5.962 (5.912 to 6.006) | <0.001 |
| Southeast Asia | 48 (-5 to 114) | 0.15 (-0.02 to 0.36) | 569 (100 to 1098) | 0.7 (0.12 to 1.34) | 5.009 (4.984 to 5.035) | <0.001 |
| Southern Latin America | 134 (30 to 260) | 2.32 (0.52 to 4.5) | 285 (72 to 518) | 2.64 (0.67 to 4.77) | 0.493 (0.373 to 0.602) | <0.001 |
| Southern Sub-Saharan Africa | 45 (11 to 87) | 1.36 (0.33 to 2.61) | 235 (63 to 422) | 3.16 (0.85 to 5.67) | 2.796 (2.733 to 2.852) | <0.001 |
| Tropical Latin America | 116 (23 to 227) | 1.09 (0.22 to 2.12) | 543 (127 to 998) | 1.69 (0.4 to 3.11) | 1.452 (1.388 to 1.528) | <0.001 |
| Western Europe | 1930 (392 to 3641) | 2.56 (0.52 to 4.82) | 2781 (640 to 5174) | 2.32 (0.54 to 4.3) | -0.332 (-0.379 to -0.287) | <0.001 |
| Western Sub-Saharan Africa | 27 (4 to 55) | 0.29 (0.04 to 0.59) | 196 (42 to 382) | 0.85 (0.18 to 1.66) | 3.483 (3.447 to 3.521) | <0.001 |

Note: DALYs, disability-adjusted life-years; BMI, body mass index; ASR, age-standardized rate; AAPC, average annual percentage change; CI, confidence interval.

**Supplement Table 3**. Mortality and DALYs of early-onset uterine cancer attributable to high BMI in 21 GBD regions, 1990-2021.

| GBD regions | Number in 1990 (95% CI) | ASR in 1990 (95% CI) | Number in 2021 (95% CI) | ASR in 2021 (95% CI) | AAPC of ASR (95% CI) | *P* |
| --- | --- | --- | --- | --- | --- | --- |
| **DALYs** |  |  |  |  |  |  |
| Global | 53498 (35988 to 73358) | 5.82 (3.92 to 7.97) | 114177 (79930 to 152290) | 6.81 (4.77 to 9.08) | 0.560 (0.490 to 0.637) | <0.001 |
| High SDI | 10327 (7245 to 13814) | 5.35 (3.75 to 7.16) | 20163 (14572 to 26015) | 8.26 (5.97 to 10.66) | 1.490 (1.395 to 1.555) | <0.001 |
| High-middle SDI | 20558 (13966 to 28149) | 10.25 (6.98 to 14.04) | 29261 (20413 to 39474) | 9.21 (6.42 to 12.44) | -0.273 (-0.368 to -0.163) | <0.001 |
| Middle SDI | 15529 (9680 to 22247) | 5.45 (3.42 to 7.78) | 38135 (25499 to 52162) | 6.79 (4.54 to 9.29) | 0.723 (0.691 to 0.750) | <0.001 |
| Low-middle SDI | 5245 (3475 to 7211) | 2.99 (1.99 to 4.1) | 19397 (13008 to 26674) | 5.09 (3.42 to 6.99) | 1.712 (1.691 to 1.730) | <0.001 |
| Low SDI | 1726 (1086 to 2495) | 2.54 (1.6 to 3.66) | 7022 (4413 to 10344) | 4.06 (2.55 to 5.97) | 1.545 (1.518 to 1.573) | <0.001 |
| Andean Latin America | 981 (622 to 1464) | 16.75 (10.64 to 25.01) | 2121 (1335 to 3227) | 15.11 (9.52 to 22.98) | -0.338 (-0.542 to -0.109) | 0.004 |
| Australasia | 199 (127 to 285) | 4.5 (2.88 to 6.43) | 447 (306 to 618) | 6.42 (4.38 to 8.88) | 1.190 (0.937 to 1.423) | <0.001 |
| Caribbean | 1166 (772 to 1614) | 18.35 (12.17 to 25.36) | 2951 (1942 to 4176) | 28.92 (19.02 to 40.94) | 1.483 (1.300 to 1.650) | <0.001 |
| Central Asia | 1719 (1172 to 2322) | 17.24 (11.76 to 23.26) | 2716 (1783 to 3846) | 13.2 (8.68 to 18.67) | -1.039 (-1.206 to -0.866) | <0.001 |
| Central Europe | 3516 (2390 to 4870) | 13.36 (9.1 to 18.49) | 3342 (2323 to 4570) | 11.69 (8.12 to 16.01) | -0.543 (-0.723 to -0.399) | <0.001 |
| Central Latin America | 1827 (1275 to 2458) | 7.24 (5.05 to 9.71) | 6367 (4455 to 8644) | 11.09 (7.76 to 15.06) | 1.496 (1.322 to 1.676) | <0.001 |
| Central Sub-Saharan Africa | 155 (86 to 261) | 2.15 (1.21 to 3.6) | 830 (426 to 1466) | 4.13 (2.12 to 7.26) | 2.124 (2.064 to 2.176) | <0.001 |
| East Asia | 12264 (6430 to 19167) | 5.53 (2.93 to 8.62) | 21725 (12480 to 34330) | 6.24 (3.58 to 9.85) | 0.408 (0.334 to 0.469) | <0.001 |
| Eastern Europe | 9394 (6395 to 12533) | 20.68 (14.09 to 27.6) | 11090 (7513 to 15012) | 21.09 (14.26 to 28.57) | 0.238 (-0.026 to 0.520) | 0.068 |
| Eastern Sub-Saharan Africa | 734 (417 to 1105) | 3.01 (1.71 to 4.53) | 2894 (1654 to 4998) | 4.45 (2.56 to 7.64) | 1.284 (1.256 to 1.309) | <0.001 |
| High-income Asia Pacific | 1063 (723 to 1500) | 2.57 (1.75 to 3.64) | 1778 (1182 to 2509) | 3.97 (2.64 to 5.61) | 1.481 (1.341 to 1.606) | <0.001 |
| High-income North America | 4324 (3048 to 5696) | 6.9 (4.87 to 9.1) | 10414 (7581 to 13213) | 13.33 (9.7 to 16.91) | 2.288 (2.116 to 2.400) | <0.001 |
| North Africa and Middle East | 2916 (1734 to 4443) | 6.11 (3.65 to 9.29) | 8774 (5959 to 12024) | 6.73 (4.57 to 9.21) | 0.332 (0.285 to 0.388) | <0.001 |
| Oceania | 113 (61 to 190) | 11.94 (6.51 to 20.04) | 407 (193 to 753) | 16.13 (7.66 to 29.87) | 1.011 (0.963 to 1.058) | <0.001 |
| South Asia | 2336 (1444 to 3554) | 1.4 (0.86 to 2.12) | 11096 (6912 to 16944) | 2.93 (1.83 to 4.45) | 2.407 (2.347 to 2.452) | <0.001 |
| Southeast Asia | 3335 (1806 to 5050) | 4.38 (2.39 to 6.59) | 12740 (7178 to 18814) | 7.98 (4.5 to 11.79) | 1.971 (1.945 to 1.996) | <0.001 |
| Southern Latin America | 974 (626 to 1398) | 10.08 (6.48 to 14.45) | 967 (661 to 1365) | 6.26 (4.28 to 8.84) | -1.427 (-1.605 to -1.219) | <0.001 |
| Southern Sub-Saharan Africa | 540 (346 to 783) | 6.71 (4.35 to 9.66) | 1658 (1083 to 2399) | 9.94 (6.51 to 14.34) | 1.320 (1.170 to 1.485) | <0.001 |
| Tropical Latin America | 1672 (1116 to 2336) | 6.51 (4.36 to 9.07) | 4382 (3043 to 5856) | 8.02 (5.57 to 10.72) | 0.550 (0.357 to 0.841) | 0.01 |
| Western Europe | 3705 (2559 to 5021) | 4.47 (3.09 to 6.06) | 5133 (3612 to 6840) | 5.23 (3.68 to 6.98) | 0.436 (0.308 to 0.531) | <0.001 |
| Western Sub-Saharan Africa | 565 (360 to 839) | 2.3 (1.47 to 3.41) | 2345 (1455 to 3574) | 3.2 (2 to 4.86) | 1.038 (0.993 to 1.070) | <0.001 |
| **Mortality** |  |  |  |  |  |  |
| Global | 1053 (708 to 1442) | 0.12 (0.08 to 0.16) | 2202 (1535 to 2936) | 0.13 (0.09 to 0.17) | 0.453 (0.399 to 0.520) | <0.001 |
| High SDI | 196 (138 to 264) | 0.1 (0.07 to 0.14) | 365 (264 to 467) | 0.15 (0.11 to 0.19) | 1.298 (1.214 to 1.365) | <0.001 |
| High-middle SDI | 404 (275 to 554) | 0.2 (0.14 to 0.28) | 555 (386 to 753) | 0.17 (0.12 to 0.24) | -0.445 (-0.534 to -0.345) | <0.001 |
| Middle SDI | 310 (194 to 443) | 0.11 (0.07 to 0.16) | 752 (507 to 1036) | 0.13 (0.09 to 0.18) | 0.611 (0.574 to 0.642) | <0.001 |
| Low-middle SDI | 106 (70 to 145) | 0.06 (0.04 to 0.08) | 386 (259 to 528) | 0.1 (0.07 to 0.14) | 1.645 (1.626 to 1.661) | <0.001 |
| Low SDI | 35 (22 to 51) | 0.05 (0.03 to 0.08) | 140 (88 to 206) | 0.08 (0.05 to 0.12) | 1.495 (1.473 to 1.520) | <0.001 |
| Andean Latin America | 20 (13 to 30) | 0.35 (0.22 to 0.52) | 42 (27 to 64) | 0.3 (0.19 to 0.46) | -0.436 (-0.643 to -0.205) | 0.001 |
| Australasia | 4 (2 to 5) | 0.09 (0.06 to 0.12) | 8 (6 to 11) | 0.12 (0.08 to 0.16) | 1.026 (0.759 to 1.279) | <0.001 |
| Caribbean | 23 (15 to 32) | 0.37 (0.24 to 0.51) | 59 (38 to 83) | 0.57 (0.38 to 0.81) | 1.468 (1.282 to 1.643) | <0.001 |
| Central Asia | 34 (23 to 46) | 0.35 (0.24 to 0.47) | 53 (35 to 75) | 0.26 (0.17 to 0.37) | -1.132 (-1.298 to -0.960) | <0.001 |
| Central Europe | 71 (48 to 97) | 0.27 (0.18 to 0.37) | 64 (45 to 88) | 0.22 (0.15 to 0.3) | -0.736 (-0.909 to -0.595) | <0.001 |
| Central Latin America | 37 (25 to 49) | 0.15 (0.1 to 0.2) | 127 (89 to 171) | 0.22 (0.15 to 0.3) | 1.411 (1.240 to 1.599) | <0.001 |
| Central Sub-Saharan Africa | 3 (2 to 5) | 0.04 (0.03 to 0.07) | 17 (9 to 30) | 0.09 (0.04 to 0.15) | 2.086 (2.025 to 2.139) | <0.001 |
| East Asia | 243 (129 to 380) | 0.11 (0.06 to 0.17) | 421 (239 to 668) | 0.12 (0.07 to 0.19) | 0.218 (0.142 to 0.280) | <0.001 |
| Eastern Europe | 183 (125 to 245) | 0.41 (0.28 to 0.54) | 209 (139 to 282) | 0.4 (0.26 to 0.53) | 0.095 (-0.191 to 0.405) | 0.432 |
| Eastern Sub-Saharan Africa | 15 (8 to 22) | 0.06 (0.04 to 0.09) | 57 (33 to 99) | 0.09 (0.05 to 0.15) | 1.233 (1.213 to 1.254) | <0.001 |
| High-income Asia Pacific | 21 (14 to 30) | 0.05 (0.03 to 0.07) | 34 (23 to 48) | 0.07 (0.05 to 0.1) | 1.284 (1.121 to 1.428) | <0.001 |
| High-income North America | 79 (56 to 104) | 0.13 (0.09 to 0.17) | 183 (134 to 229) | 0.23 (0.17 to 0.29) | 1.984 (1.816 to 2.205) | <0.001 |
| North Africa and Middle East | 58 (34 to 88) | 0.12 (0.07 to 0.19) | 166 (113 to 227) | 0.13 (0.09 to 0.17) | 0.149 (0.101 to 0.206) | <0.001 |
| Oceania | 2 (1 to 4) | 0.24 (0.13 to 0.41) | 8 (4 to 15) | 0.33 (0.16 to 0.61) | 1.001 (0.952 to 1.050) | <0.001 |
| South Asia | 47 (29 to 72) | 0.03 (0.02 to 0.04) | 221 (138 to 335) | 0.06 (0.04 to 0.09) | 2.313 (2.253 to 2.358) | <0.001 |
| Southeast Asia | 66 (36 to 100) | 0.09 (0.05 to 0.13) | 253 (142 to 375) | 0.16 (0.09 to 0.23) | 1.908 (1.880 to 1.931) | <0.001 |
| Southern Latin America | 19 (12 to 28) | 0.2 (0.13 to 0.29) | 19 (13 to 26) | 0.12 (0.08 to 0.17) | -1.576 (-1.750 to -1.368) | <0.001 |
| Southern Sub-Saharan Africa | 11 (7 to 16) | 0.14 (0.09 to 0.2) | 33 (22 to 48) | 0.2 (0.13 to 0.29) | 1.281 (1.108 to 1.442) | <0.001 |
| Tropical Latin America | 34 (23 to 48) | 0.14 (0.09 to 0.19) | 87 (61 to 116) | 0.16 (0.11 to 0.21) | 0.403 (0.209 to 0.685) | 0.011 |
| Western Europe | 71 (49 to 96) | 0.09 (0.06 to 0.12) | 92 (65 to 123) | 0.09 (0.07 to 0.12) | 0.209 (0.099 to 0.297) | <0.001 |
| Western Sub-Saharan Africa | 11 (7 to 17) | 0.05 (0.03 to 0.07) | 47 (29 to 71) | 0.07 (0.04 to 0.1) | 0.983 (0.936 to 1.016) | <0.001 |

Note: DALYs, disability-adjusted life-years; BMI, body mass index; ASR, age-standardized rate; AAPC, average annual percentage change; CI, confidence interval.

**Supplement Table 4**. Mortality and DALYs of late-onset uterine cancer attributable to high BMI in 21 GBD regions, 1990-2021.

| GBD regions | Number in 1990 (95% CI) | ASR in 1990 (95% CI) | Number in 2021 (95% CI) | ASR in 2021 (95% CI) | AAPC of ASR (95%CI) | *P* |
| --- | --- | --- | --- | --- | --- | --- |
| DALYs |  |  |  |  |  |  |
| Global | 319143 (224866 to 427531) | 67.41 (47.49 to 90.28) | 765969 (544681 to 1006894) | 74.43 (53.03 to 97.78) | 0.300 (0.254 to 0.342) | <0.001 |
| High SDI | 120949 (85574 to 161987) | 86.59 (61.43 to 115.62) | 263993 (188268 to 343022) | 110.73 (79.77 to 143.09) | 0.800 (0.761 to 0.835) | <0.001 |
| High-middle SDI | 130454 (91699 to 173806) | 103.05 (72.47 to 137.3) | 240727 (169417 to 318311) | 98.95 (69.71 to 130.81) | -0.110 (-0.177 to -0.031) | 0.01 |
| Middle SDI | 43768 (29427 to 60819) | 36.63 (24.63 to 50.89) | 164031 (112768 to 225808) | 50.55 (34.76 to 69.57) | 1.039 (0.997 to 1.076) | <0.001 |
| Low-middle SDI | 16970 (11595 to 23502) | 25.21 (17.22 to 34.92) | 73887 (49671 to 101321) | 43.96 (29.55 to 60.31) | 1.817 (1.791 to 1.840) | <0.001 |
| Low SDI | 6335 (4091 to 9164) | 25.12 (16.22 to 36.37) | 21985 (14078 to 31954) | 38.07 (24.38 to 55.35) | 1.362 (1.354 to 1.370) | <0.001 |
| Andean Latin America | 2793 (1772 to 4076) | 122.37 (77.64 to 178.73) | 8379 (5194 to 12923) | 123.9 (76.84 to 191.11) | 0.139 (-0.037 to 0.342) | 0.114 |
| Australasia | 1895 (1266 to 2667) | 67.7 (45.22 to 95.02) | 5598 (3804 to 7706) | 91.29 (62.43 to 125.31) | 0.989 (0.796 to 1.110) | <0.001 |
| Caribbean | 3112 (2123 to 4275) | 107.23 (73.14 to 147.28) | 12056 (8270 to 16726) | 190.93 (131.14 to 264.75) | 2.030 (1.896 to 2.184) | <0.001 |
| Central Asia | 8844 (6194 to 11735) | 141.53 (99.19 to 187.87) | 12639 (8810 to 17073) | 118.47 (82.58 to 160.12) | -0.632 (-0.763 to -0.522) | <0.001 |
| Central Europe | 29600 (20645 to 39278) | 152.13 (106.19 to 201.94) | 48367 (34239 to 64661) | 175.25 (124.29 to 233.88) | 0.402 (0.309 to 0.468) | <0.001 |
| Central Latin America | 6444 (4475 to 8694) | 68.64 (47.64 to 92.67) | 28852 (20060 to 39217) | 95.42 (66.37 to 129.69) | 1.194 (1.078 to 1.308) | <0.001 |
| Central Sub-Saharan Africa | 863 (482 to 1429) | 30.07 (16.78 to 49.93) | 3928 (2136 to 6710) | 57.33 (31.1 to 98.32) | 2.122 (2.097 to 2.146) | <0.001 |
| East Asia | 28221 (17274 to 43147) | 27.98 (17.12 to 42.79) | 93680 (56099 to 146930) | 35.59 (21.32 to 55.85) | 0.776 (0.713 to 0.834) | <0.001 |
| Eastern Europe | 74175 (52119 to 98112) | 183.08 (128.73 to 241.93) | 112117 (78681 to 148309) | 236.23 (166.05 to 312.5) | 0.840 (0.644 to 1.037) | <0.001 |
| Eastern Sub-Saharan Africa | 2569 (1437 to 3809) | 30.37 (17 to 45.03) | 9248 (5506 to 14351) | 47.37 (28.2 to 73.46) | 1.452 (1.438 to 1.468) | <0.001 |
| High-income Asia Pacific | 6729 (4677 to 9258) | 26.62 (18.5 to 36.62) | 15122 (9882 to 21233) | 34.27 (22.88 to 47.85) | 0.811 (0.724 to 0.874) | <0.001 |
| High-income North America | 49558 (34925 to 66410) | 114.17 (80.89 to 152.08) | 137329 (98712 to 175215) | 176.43 (127.61 to 223.98) | 1.419 (1.355 to 1.476) | <0.001 |
| North Africa and Middle East | 9446 (5977 to 14017) | 50.4 (31.87 to 74.81) | 32599 (21498 to 44968) | 64.77 (42.64 to 89.43) | 0.767 (0.717 to 0.828) | <0.001 |
| Oceania | 318 (179 to 523) | 94.79 (53.59 to 155.23) | 1101 (572 to 1805) | 129.43 (67.7 to 210.05) | 1.026 (0.971 to 1.081) | <0.001 |
| South Asia | 6774 (4119 to 10431) | 10.98 (6.67 to 16.92) | 41737 (25675 to 62944) | 24.62 (15.14 to 37.15) | 2.670 (2.627 to 2.711) | <0.001 |
| Southeast Asia | 7390 (4506 to 10751) | 23.62 (14.43 to 34.35) | 39519 (23694 to 57095) | 47.79 (28.68 to 69.05) | 2.318 (2.290 to 2.346) | <0.001 |
| Southern Latin America | 5915 (3904 to 8327) | 103.25 (68.09 to 145.29) | 9300 (6335 to 12728) | 87.62 (59.92 to 119.56) | -0.492 (-0.569 to -0.417) | <0.001 |
| Southern Sub-Saharan Africa | 2089 (1308 to 3138) | 62.24 (38.97 to 93.52) | 9647 (6191 to 13250) | 128.23 (82.17 to 176.21) | 2.324 (2.179 to 2.451) | <0.001 |
| Tropical Latin America | 9163 (6200 to 12786) | 85.25 (57.62 to 119.05) | 29905 (20675 to 40300) | 94.59 (65.51 to 127.34) | 0.333 (0.269 to 0.422) | <0.001 |
| Western Europe | 60218 (42026 to 81333) | 81.43 (56.96 to 109.78) | 102295 (70827 to 138063) | 94.34 (66.17 to 126.36) | 0.431 (0.367 to 0.486) | <0.001 |
| Western Sub-Saharan Africa | 3028 (1936 to 4526) | 32.3 (20.64 to 48.32) | 12551 (7843 to 18199) | 54.75 (34.24 to 79.2) | 1.695 (1.677 to 1.709) | <0.001 |
| Mortality |  |  |  |  |  |  |
| Global | 12841 (9030 to 17245) | 2.67 (1.88 to 3.58) | 30933 (21783 to 40832) | 2.87 (2.03 to 3.79) | 0.219 (0.173 to 0.257) | <0.001 |
| High SDI | 5305 (3714 to 7175) | 3.45 (2.43 to 4.65) | 11473 (7990 to 15108) | 4.17 (2.95 to 5.44) | 0.615 (0.579 to 0.647) | <0.001 |
| High-middle SDI | 5024 (3521 to 6714) | 3.88 (2.72 to 5.19) | 9659 (6718 to 12794) | 3.75 (2.61 to 4.96) | -0.081 (-0.154 to 0.011) | 0.072 |
| Middle SDI | 1620 (1091 to 2250) | 1.39 (0.94 to 1.94) | 6147 (4194 to 8483) | 1.89 (1.29 to 2.61) | 0.991 (0.948 to 1.030) | <0.001 |
| Low-middle SDI | 634 (433 to 880) | 0.98 (0.67 to 1.37) | 2788 (1872 to 3843) | 1.68 (1.13 to 2.32) | 1.755 (1.713 to 1.785) | <0.001 |
| Low SDI | 229 (148 to 332) | 0.95 (0.61 to 1.38) | 807 (516 to 1173) | 1.45 (0.93 to 2.11) | 1.393 (1.383 to 1.404) | <0.001 |
| Andean Latin America | 106 (67 to 155) | 4.65 (2.94 to 6.85) | 332 (205 to 514) | 4.8 (2.96 to 7.41) | 0.198 (0.018 to 0.420) | 0.034 |
| Australasia | 82 (55 to 117) | 2.68 (1.78 to 3.8) | 251 (168 to 346) | 3.57 (2.41 to 4.88) | 0.959 (0.785 to 1.075) | <0.001 |
| Caribbean | 122 (83 to 168) | 4.1 (2.79 to 5.64) | 491 (334 to 682) | 7.43 (5.06 to 10.3) | 2.091 (1.959 to 2.240) | <0.001 |
| Central Asia | 330 (230 to 438) | 5.26 (3.67 to 7) | 463 (322 to 627) | 4.47 (3.11 to 6.05) | -0.568 (-0.706 to -0.451) | <0.001 |
| Central Europe | 1212 (841 to 1610) | 6.03 (4.19 to 8.01) | 2200 (1536 to 2948) | 7.02 (4.92 to 9.38) | 0.466 (0.413 to 0.513) | <0.001 |
| Central Latin America | 254 (175 to 345) | 2.75 (1.9 to 3.73) | 1111 (770 to 1513) | 3.62 (2.51 to 4.93) | 1.022 (0.922 to 1.124) | <0.001 |
| Central Sub-Saharan Africa | 30 (17 to 50) | 1.13 (0.63 to 1.89) | 140 (76 to 241) | 2.18 (1.17 to 3.78) | 2.155 (2.131 to 2.179) | <0.001 |
| East Asia | 1003 (619 to 1532) | 1.01 (0.62 to 1.54) | 3358 (2012 to 5243) | 1.26 (0.75 to 1.96) | 0.706 (0.643 to 0.764) | <0.001 |
| Eastern Europe | 2792 (1954 to 3700) | 6.64 (4.65 to 8.79) | 4416 (3085 to 5856) | 8.59 (6 to 11.38) | 0.843 (0.669 to 1.020) | <0.001 |
| Eastern Sub-Saharan Africa | 93 (52 to 138) | 1.15 (0.65 to 1.7) | 342 (203 to 529) | 1.83 (1.09 to 2.83) | 1.522 (1.508 to 1.539) | <0.001 |
| High-income Asia Pacific | 280 (193 to 386) | 1.06 (0.73 to 1.46) | 699 (440 to 991) | 1.26 (0.82 to 1.76) | 0.487 (0.405 to 0.563) | <0.001 |
| High-income North America | 2115 (1460 to 2859) | 4.35 (3.03 to 5.83) | 5517 (3878 to 7134) | 6.42 (4.56 to 8.23) | 1.261 (1.198 to 1.310) | <0.001 |
| North Africa and Middle East | 348 (219 to 518) | 1.95 (1.22 to 2.9) | 1201 (786 to 1660) | 2.49 (1.62 to 3.45) | 0.763 (0.702 to 0.832) | <0.001 |
| Oceania | 11 (6 to 18) | 3.4 (1.94 to 5.49) | 37 (20 to 60) | 4.68 (2.47 to 7.48) | 1.049 (1.000 to 1.089) | <0.001 |
| South Asia | 250 (151 to 385) | 0.42 (0.26 to 0.66) | 1578 (969 to 2382) | 0.94 (0.58 to 1.42) | 2.626 (2.567 to 2.676) | <0.001 |
| Southeast Asia | 251 (154 to 365) | 0.83 (0.51 to 1.2) | 1366 (824 to 1978) | 1.68 (1.02 to 2.44) | 2.334 (2.305 to 2.363) | <0.001 |
| Southern Latin America | 248 (164 to 350) | 4.18 (2.76 to 5.9) | 409 (275 to 562) | 3.55 (2.4 to 4.85) | -0.497 (-0.583 to -0.412) | <0.001 |
| Southern Sub-Saharan Africa | 84 (53 to 127) | 2.52 (1.57 to 3.81) | 390 (247 to 538) | 5.27 (3.32 to 7.27) | 2.385 (2.256 to 2.500) | <0.001 |
| Tropical Latin America | 364 (244 to 510) | 3.45 (2.31 to 4.83) | 1228 (839 to 1660) | 3.76 (2.58 to 5.08) | 0.294 (0.232 to 0.378) | <0.001 |
| Western Europe | 2747 (1896 to 3732) | 3.29 (2.28 to 4.47) | 4918 (3333 to 6709) | 3.74 (2.58 to 5.04) | 0.381 (0.336 to 0.423) | <0.001 |
| Western Sub-Saharan Africa | 118 (75 to 178) | 1.27 (0.81 to 1.92) | 484 (302 to 699) | 2.25 (1.4 to 3.24) | 1.846 (1.830 to 1.859) | <0.001 |

Note: DALYs, disability-adjusted life-years; BMI, body mass index; ASR, age-standardized rate; AAPC, average annual percentage change; CI, confidence interval.

**Supplement Table 5**. The top ten countries with the highest ASR for early-onset/late-onset ovarian cancer and uterine cancer attributable to high BMI in 2021 (retained countries with very small case numbers).

| Cancer | Countries | Number in 1990 (95% CI) | ASR in 1990 (95% CI) | Number in 2021 (95% CI) | ASR in 2021 (95%CI) | AAPC of ASR (95% CI) | *P* |
| --- | --- | --- | --- | --- | --- | --- | --- |
| **DALYs** |  |  |  |  |  |  |  |
| Early-onset ovarian cancer | Bahamas | 9 (2 to 19) | 18.48 (3.66 to 37.72) | 29 (7 to 55) | 30.81 (7.46 to 57.91) | 1.6 (1.5 to 1.7) | <0.001 |
|  | Seychelles | 2 (0 to 3) | 14.11 (2.84 to 28.98) | 7 (2 to 13) | 29.44 (7.18 to 59.4) | 2.8 (2.4 to 3.2) | <0.001 |
|  | United Arab Emirates | 25 (4 to 64) | 12.74 (2.13 to 32.23) | 448 (117 to 922) | 24.68 (6.44 to 51.18) | 2.3 (2.2 to 2.4) | <0.001 |
|  | Grenada | 2 (0 to 3) | 12.73 (2.16 to 28.06) | 5 (1 to 10) | 24.19 (6.05 to 45.78) | 2.1 (2 to 2.2) | <0.001 |
|  | Trinidad and Tobago | 34 (7 to 66) | 15.52 (3.2 to 30.08) | 76 (20 to 150) | 23.86 (6.25 to 46.99) | 1.5 (1.4 to 1.7) | <0.001 |
|  | United States Virgin Islands | 4 (1 to 9) | 15.94 (3.69 to 34.25) | 4 (1 to 8) | 23.71 (5.78 to 51.34) | 1.4 (1.2 to 1.6) | <0.001 |
|  | Saint Lucia | 3 (1 to 6) | 14.45 (2.88 to 29.17) | 10 (3 to 18) | 22.95 (6.2 to 42.04) | 1.6 (1.4 to 1.7) | <0.001 |
|  | Guyana | 11 (2 to 25) | 9.24 (1.46 to 19.55) | 34 (8 to 67) | 21.18 (5.1 to 41.79) | 2.8 (2.6 to 2.9) | <0.001 |
|  | Mexico | 1217 (264 to 2290) | 8.98 (1.99 to 16.76) | 6032 (1713 to 10992) | 19.98 (5.66 to 36.41) | 2.7 (2.5 to 2.8) | <0.001 |
|  | Greenland | 2 (0 to 5) | 20.62 (4.35 to 43.65) | 2 (0 to 4) | 19.51 (3.78 to 41.91) | -0.1 (-0.2 to 0) | 0.191 |
| Late-onset ovarian cancer | United Arab Emirates | 28 (4 to 73) | 88.8 (14.11 to 232.2) | 461 (126 to 931) | 309.07 (79.39 to 611.25) | 3.977 (3.75 to 4.209) | <0.001 |
|  | Bahrain | 14 (3 to 30) | 77.5 (14.72 to 160.7) | 124 (37 to 246) | 134.36 (38.91 to 266.36) | 1.828 (1.728 to 1.935) | <0.001 |
|  | Latvia | 399 (94 to 753) | 82.61 (19.55 to 155.73) | 569 (140 to 1082) | 126.26 (31.13 to 239.66) | 1.429 (1.199 to 1.685) | <0.001 |
|  | Qatar | 5 (1 to 11) | 66.58 (13.29 to 146.54) | 83 (23 to 168) | 122.17 (34.44 to 244.54) | 2.097 (1.897 to 2.304) | <0.001 |
|  | Serbia | 938 (195 to 1898) | 64.37 (13.33 to 130.9) | 2082 (564 to 3912) | 114.27 (30.62 to 215.67) | 1.79 (1.685 to 1.903) | <0.001 |
|  | Eswatini | 20 (4 to 46) | 56.86 (11.96 to 132.67) | 83 (20 to 180) | 111.22 (26.73 to 241.8) | 2.156 (2.069 to 2.248) | <0.001 |
|  | Poland | 4978 (1136 to 9275) | 91.1 (20.82 to 169.75) | 9273 (2305 to 17136) | 109.98 (27.33 to 203.27) | 0.563 (0.48 to 0.644) | <0.001 |
|  | Libya | 83 (16 to 176) | 42.42 (8.35 to 90.04) | 664 (179 to 1321) | 109.7 (29.51 to 219.41) | 3.118 (3.088 to 3.148) | <0.001 |
|  | Georgia | 181 (41 to 360) | 21.45 (4.84 to 42.82) | 794 (183 to 1555) | 108.78 (25.03 to 212.36) | 5.565 (5.245 to 5.841) | <0.001 |
|  | Bahamas | 13 (3 to 25) | 68.49 (14.49 to 136.9) | 57 (14 to 106) | 107.93 (26.83 to 201.49) | 1.538 (1.438 to 1.664) | <0.001 |
| Early-onset uterine cancer | Nauru | 1 (0 to 1) | 37.72 (12.68 to 78.31) | 1 (0 to 2) | 50.27 (18.25 to 102.81) | 0.893 (0.862 to 0.934) | <0.001 |
|  | Marshall Islands | 1 (1 to 3) | 27.94 (13.84 to 49.62) | 6 (2 to 13) | 49.11 (17.44 to 113.86) | 1.831 (1.82 to 1.84) | <0.001 |
|  | Northern Mariana Islands | 3 (1 to 5) | 32.54 (14.75 to 63.65) | 6 (4 to 10) | 48.41 (26.87 to 80.77) | 1.128 (0.794 to 1.423) | <0.001 |
|  | American Samoa | 2 (1 to 3) | 24.89 (12.38 to 44.75) | 5 (2 to 10) | 44.58 (17.81 to 93.71) | 1.955 (1.879 to 2.026) | <0.001 |
|  | Bahamas | 13 (9 to 19) | 27.46 (17.6 to 38.81) | 42 (26 to 61) | 43.85 (27.24 to 63.73) | 1.426 (1.267 to 1.576) | <0.001 |
|  | Micronesia (Federated States of) | 4 (2 to 7) | 28.72 (11.88 to 55.24) | 8 (4 to 16) | 40.59 (17.98 to 81.22) | 1.121 (1.11 to 1.133) | <0.001 |
|  | Guyana | 22 (12 to 35) | 18.12 (10.23 to 28.92) | 62 (35 to 98) | 39.08 (22.08 to 62.17) | 2.476 (2.208 to 2.747) | <0.001 |
|  | Trinidad and Tobago | 53 (34 to 72) | 24.48 (16 to 33.54) | 125 (74 to 187) | 38.68 (22.97 to 58.18) | 1.439 (1.192 to 1.677) | <0.001 |
|  | Barbados | 13 (8 to 18) | 25.7 (16.82 to 35.78) | 26 (17 to 38) | 36.85 (23.59 to 54.18) | 1.356 (1.177 to 1.565) | <0.001 |
|  | Jamaica | 52 (33 to 76) | 14.9 (9.43 to 21.62) | 222 (126 to 350) | 35.6 (20.3 to 56.13) | 2.894 (2.481 to 3.249) | <0.001 |
| Late-onset uterine cancer | United Arab Emirates | 65 (27 to 127) | 213.21 (89.03 to 414.76) | 583 (330 to 974) | 464.14 (265.58 to 764.6) | 2.412 (2.157 to 2.651) | <0.001 |
|  | Northern Mariana Islands | 3 (2 to 7) | 233.59 (107.9 to 452.91) | 23 (13 to 37) | 358.12 (202.33 to 578.33) | 1.418 (1.303 to 1.539) | <0.001 |
|  | Nauru | 1 (0 to 2) | 241.45 (101.79 to 469.23) | 2 (1 to 4) | 306.25 (134.92 to 578.09) | 0.753 (0.735 to 0.773) | <0.001 |
|  | Marshall Islands | 3 (2 to 6) | 180.01 (88.45 to 321.85) | 12 (5 to 29) | 285.48 (106.61 to 674.84) | 1.487 (1.477 to 1.498) | <0.001 |
|  | Honduras | 323 (168 to 558) | 140.68 (73.02 to 243.26) | 2152 (1047 to 3740) | 284.58 (138.39 to 494.86) | 2.36 (2.229 to 2.47) | <0.001 |
|  | Barbados | 55 (36 to 78) | 165.58 (107.97 to 232.63) | 182 (117 to 262) | 284.37 (183.56 to 409.09) | 1.84 (1.682 to 1.999) | <0.001 |
|  | Jamaica | 210 (131 to 313) | 106.41 (66.98 to 158.14) | 958 (587 to 1453) | 276.16 (169.26 to 418.6) | 3.221 (2.998 to 3.428) | <0.001 |
|  | American Samoa | 4 (2 to 7) | 170.28 (87.77 to 288.22) | 15 (6 to 32) | 270.31 (109.71 to 549.98) | 1.538 (1.47 to 1.598) | <0.001 |
|  | Georgia | 2051 (1392 to 2794) | 237.49 (161.31 to 323.4) | 1950 (1285 to 2763) | 255.82 (168.61 to 362.12) | 0.082 (-0.274 to 0.464) | 0.707 |
|  | Trinidad and Tobago | 159 (103 to 224) | 169.74 (109.92 to 239.05) | 582 (367 to 850) | 253.3 (159.5 to 370.49) | 1.373 (1.167 to 1.585) | <0.001 |
| **Mortality** |  |  |  |  |  |  |  |
| Early-onset ovarian cancer | Bahamas | 0 (0 to 0) | 0.37 (0.07 to 0.75) | 1 (0 to 1) | 0.61 (0.15 to 1.15) | 1.6 (1.5 to 1.8) | <0.001 |
|  | Seychelles | 0 (0 to 0) | 0.28 (0.06 to 0.58) | 0 (0 to 0) | 0.56 (0.14 to 1.13) | 2.7 (2.3 to 3.2) | <0.001 |
|  | Grenada | 0 (0 to 0) | 0.26 (0.04 to 0.56) | 0 (0 to 0) | 0.48 (0.12 to 0.91) | 2.1 (2 to 2.2) | <0.001 |
|  | Trinidad and Tobago | 1 (0 to 1) | 0.31 (0.06 to 0.61) | 2 (0 to 3) | 0.48 (0.13 to 0.95) | 1.5 (1.4 to 1.7) | <0.001 |
|  | United Arab Emirates | 0 (0 to 1) | 0.26 (0.04 to 0.66) | 9 (2 to 18) | 0.48 (0.13 to 0.99) | 2.1 (2 to 2.2) | <0.001 |
|  | Saint Lucia | 0 (0 to 0) | 0.3 (0.06 to 0.6) | 0 (0 to 0) | 0.46 (0.13 to 0.85) | 1.5 (1.4 to 1.7) | <0.001 |
|  | United States Virgin Islands | 0 (0 to 0) | 0.32 (0.07 to 0.68) | 0 (0 to 0) | 0.46 (0.11 to 0.99) | 1.2 (1.1 to 1.3) | <0.001 |
|  | Guyana | 0 (0 to 0) | 0.19 (0.03 to 0.39) | 1 (0 to 1) | 0.43 (0.1 to 0.84) | 2.8 (2.6 to 2.9) | <0.001 |
|  | Greenland | 0 (0 to 0) | 0.42 (0.09 to 0.87) | 0 (0 to 0) | 0.41 (0.08 to 0.86) | 0.1 (0 to 0.2) | 0.124 |
|  | Mexico | 24 (5 to 45) | 0.18 (0.04 to 0.34) | 122 (35 to 221) | 0.4 (0.12 to 0.73) | 2.7 (2.6 to 2.8) | <0.001 |
| Late-onset ovarian cancer | United Arab Emirates | 1 (0 to 3) | 3.28 (0.52 to 8.52) | 17 (4 to 33) | 15.19 (3.81 to 30.02) | 5.162 (4.925 to 5.38) | <0.001 |
|  | Bahrain | 0 (0 to 1) | 2.9 (0.54 to 6.03) | 4 (1 to 9) | 5.34 (1.51 to 10.65) | 2.037 (1.931 to 2.153) | <0.001 |
|  | Qatar | 0 (0 to 0) | 2.61 (0.52 to 5.7) | 3 (1 to 6) | 4.99 (1.4 to 9.95) | 2.269 (2.059 to 2.473) | <0.001 |
|  | Latvia | 15 (4 to 29) | 2.98 (0.7 to 5.64) | 25 (6 to 47) | 4.71 (1.16 to 8.97) | 1.561 (1.403 to 1.735) | <0.001 |
|  | Poland | 193 (44 to 361) | 3.36 (0.77 to 6.28) | 416 (102 to 771) | 4.4 (1.09 to 8.14) | 0.811 (0.732 to 0.888) | <0.001 |
|  | Eswatini | 1 (0 to 2) | 2.23 (0.46 to 5.21) | 3 (1 to 7) | 4.37 (1.04 to 9.42) | 2.195 (2.14 to 2.252) | <0.001 |
|  | Serbia | 34 (7 to 69) | 2.41 (0.49 to 4.93) | 87 (23 to 164) | 4.35 (1.17 to 8.21) | 1.906 (1.798 to 2.033) | <0.001 |
|  | Libya | 3 (1 to 6) | 1.54 (0.29 to 3.31) | 23 (6 to 47) | 4.11 (1.09 to 8.31) | 3.206 (3.171 to 3.242) | <0.001 |
|  | Georgia | 6 (1 to 13) | 0.74 (0.17 to 1.48) | 32 (7 to 62) | 4.07 (0.93 to 7.98) | 5.819 (5.437 to 6.136) | <0.001 |
|  | Bahamas | 0 (0 to 1) | 2.43 (0.5 to 4.91) | 2 (0 to 4) | 3.97 (0.96 to 7.43) | 1.615 (1.515 to 1.718) | <0.001 |
| Early-onset uterine cancer | Nauru | 0 (0 to 0) | 0.77 (0.26 to 1.59) | 0 (0 to 0) | 1.01 (0.37 to 2.07) | 0.89 (0.85 to 0.92) | <0.001 |
|  | Marshall Islands | 0 (0 to 0) | 0.57 (0.28 to 1.02) | 0 (0 to 0) | 1 (0.36 to 2.31) | 1.79 (1.781 to 1.799) | <0.001 |
|  | Northern Mariana Islands | 0 (0 to 0) | 0.65 (0.29 to 1.26) | 0 (0 to 0) | 0.94 (0.52 to 1.56) | 1.043 (0.671 to 1.419) | <0.001 |
|  | American Samoa | 0 (0 to 0) | 0.5 (0.25 to 0.9) | 0 (0 to 0) | 0.89 (0.36 to 1.87) | 1.887 (1.817 to 1.951) | <0.001 |
|  | Bahamas | 0 (0 to 0) | 0.54 (0.35 to 0.76) | 1 (1 to 1) | 0.86 (0.54 to 1.25) | 1.383 (1.215 to 1.537) | <0.001 |
|  | Micronesia (Federated States of) | 0 (0 to 0) | 0.59 (0.24 to 1.13) | 0 (0 to 0) | 0.82 (0.37 to 1.64) | 1.079 (1.063 to 1.095) | <0.001 |
|  | Guyana | 0 (0 to 1) | 0.37 (0.21 to 0.59) | 1 (1 to 2) | 0.78 (0.44 to 1.24) | 2.456 (2.208 to 2.689) | <0.001 |
|  | Trinidad and Tobago | 1 (1 to 1) | 0.5 (0.32 to 0.68) | 2 (1 to 4) | 0.77 (0.45 to 1.15) | 1.411 (1.168 to 1.654) | <0.001 |
|  | Barbados | 0 (0 to 0) | 0.51 (0.33 to 0.71) | 1 (0 to 1) | 0.72 (0.46 to 1.06) | 1.325 (1.133 to 1.557) | <0.001 |
|  | Kiribati | 0 (0 to 0) | 0.53 (0.19 to 1) | 0 (0 to 0) | 0.72 (0.24 to 1.45) | 0.986 (0.972 to 0.999) | <0.001 |
| Late-onset uterine cancer | United Arab Emirates | 2 (1 to 4) | 7.84 (3.36 to 15.23) | 21 (12 to 34) | 22.32 (12.7 to 36.75) | 3.317 (3.011 to 3.618) | <0.001 |
|  | Northern Mariana Islands | 0 (0 to 0) | 8.34 (3.85 to 16.33) | 1 (0 to 1) | 13.05 (7.37 to 20.89) | 1.471 (1.374 to 1.582) | <0.001 |
|  | Barbados | 2 (2 to 3) | 6.44 (4.16 to 9.12) | 8 (5 to 11) | 11.5 (7.38 to 16.58) | 1.972 (1.823 to 2.121) | <0.001 |
|  | Nauru | 0 (0 to 0) | 8.66 (3.69 to 16.79) | 0 (0 to 0) | 11.1 (4.92 to 20.76) | 0.792 (0.774 to 0.811) | <0.001 |
|  | Honduras | 12 (6 to 21) | 5.34 (2.76 to 9.34) | 82 (39 to 143) | 11.01 (5.3 to 19.24) | 2.4 (2.251 to 2.557) | <0.001 |
|  | Jamaica | 9 (5 to 13) | 4.07 (2.56 to 6.12) | 39 (24 to 59) | 10.76 (6.56 to 16.27) | 3.301 (3.005 to 3.617) | <0.001 |
|  | Marshall Islands | 0 (0 to 0) | 6.59 (3.21 to 11.83) | 0 (0 to 1) | 10.41 (3.88 to 24.77) | 1.474 (1.464 to 1.483) | <0.001 |
|  | Saint Kitts and Nevis | 0 (0 to 0) | 6.94 (4.38 to 10.12) | 1 (1 to 1) | 9.99 (6.47 to 14.18) | 1.274 (1.064 to 1.556) | <0.001 |
|  | American Samoa | 0 (0 to 0) | 6.22 (3.18 to 10.56) | 1 (0 to 1) | 9.85 (3.99 to 20.13) | 1.542 (1.485 to 1.599) | <0.001 |
|  | Georgia | 77 (52 to 105) | 8.74 (5.89 to 11.94) | 80 (53 to 113) | 9.75 (6.46 to 13.79) | 0.218 (-0.153 to 0.616) | 0.266 |

Note: DALYs, disability-adjusted life-years; BMI, body mass index; ASR, age-standardized rate; AAPC, average annual percentage change; CI, confidence interval.


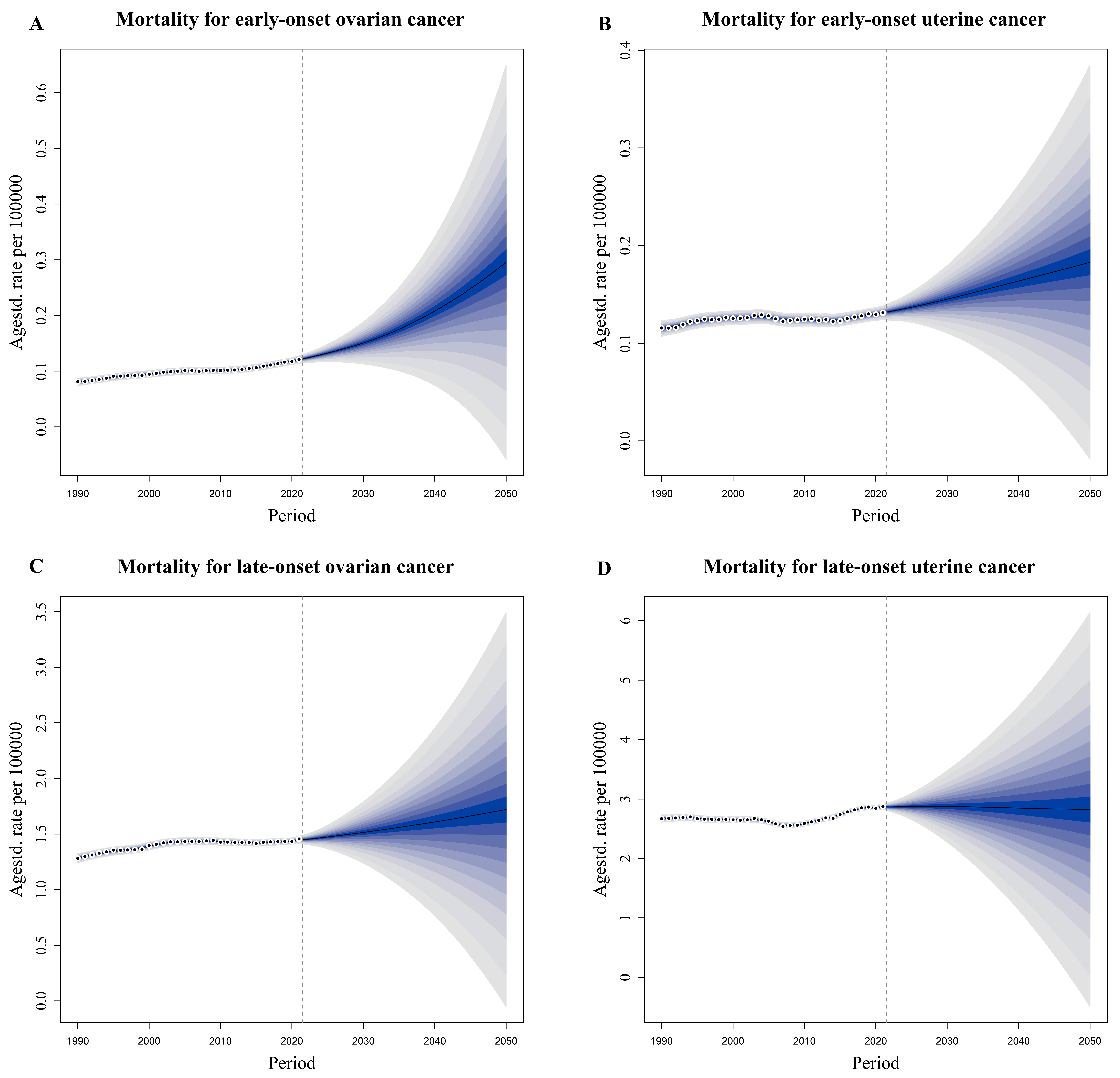


**Supplement Figure 1**. The predictive trends of ASMR in ovarian cancer and uterine cancer attributable to high BMI from 2022 to 2050. (A) early-onset ovarian cancer; (B) early-onset uterine cancer; (C) late-onset ovarian cancer; (D) late-onset uterine cancer. ASMR, age-standardized mortality rates; BMI, body mass index.
